# Rhinovirus as a driver of airway T cell dynamics in children with treatment-refractory recurrent wheeze

**DOI:** 10.1172/jci.insight.189480

**Published:** 2025-05-08

**Authors:** Naomi Bryant, Lyndsey M. Muehling, Kristin Wavell, W. Gerald Teague, Judith A. Woodfolk

**Affiliations:** 1 Department of Medicine,; 2 Department of Microbiology, Immunology, and Cancer Biology, and; 3 Child Health Research Center, Department of Pediatrics, University of Virginia School of Medicine, Charlottesville, Virginia, USA.

**Keywords:** Immunology, Infectious disease, T cells, Th1 response

## Abstract

Severe asthma in children is notoriously difficult to treat, and its immunopathogenesis is complex. In particular, the contribution of T cells and relationships to antiviral immunity remain enigmatic. Here, we coupled deep phenotyping with machine learning methods to elucidate the dynamics of T cells in the lower airways of children with treatment-refractory recurrent wheeze, and examine rhinovirus (RV) as a driver. Our strategy revealed a T cell landscape dominated by type 1 and type 17 CD8^+^ signatures. Interrogation of phenotypic relationships coupled with trajectory mapping identified T cell migratory and differentiation pathways spanning the blood and airways that culminated in tissue residency, and involved transitions between type 1 and type 17 tissue-resident types. These dynamics were reflected in cytokine polyfunctionality. Use of machine learning tools to cross-compare T cell populations that were enriched in the airways of RV-positive children with those induced in the blood following experimental RV challenge precisely pinpointed RV-responsive signatures that contributed to T cell migratory and differentiation pathways. Despite their rarity, these signatures were also detected in the airways of RV-negative children. Together, our results underscore the aberrant nature of type 1 immunity in the airways of children with recurrent wheeze, and implicate an important viral trigger as a driver.

## Abstract 2

This study establishes a link between rhinovirus, an important trigger of acute wheeze, and aberrant airway T-cell dynamics in children with treatment-refractory recurrent wheeze.

## Graphical abstract

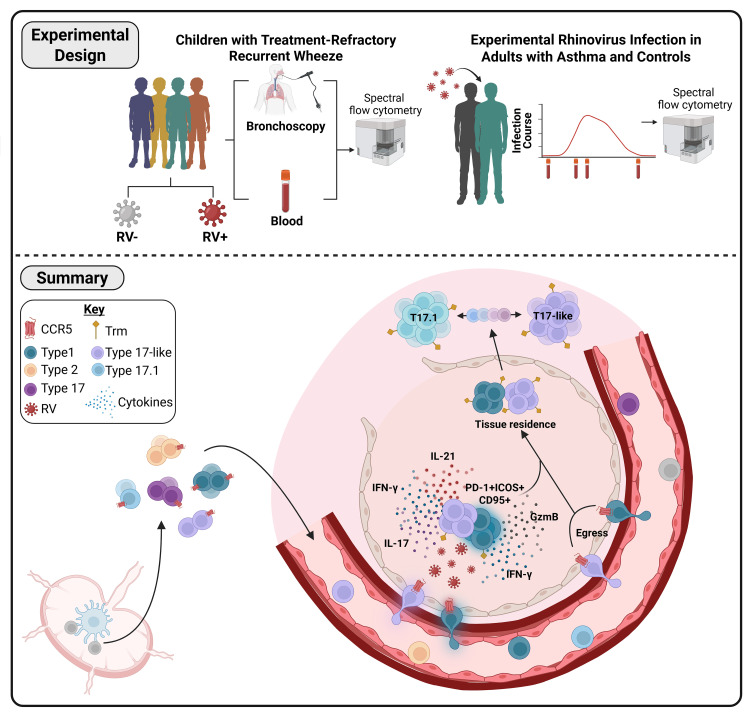

## Introduction

Asthma affects 7% of children in the United States, amounting to approximately 4.6 million cases (1). Its characteristic recurrent episodes of cough, wheeze, and shortness of breath reflect underlying chronic inflammatory processes in the lower airways and lung parenchyma (2, 3). While many children who undergo treatment attain symptom control with minimal exacerbations, approximately 5%–10% of cases are severe and remain refractory to standard therapies, including inhaled corticosteroids (ICS) (3). Consequently, patients with severe disease experience frequent acute exacerbations, often requiring hospitalization, and thus, account for a disproportionate amount of the enormous health and economic burden of asthma (4–6). Despite the advent of biologic therapies that target type 2 inflammatory pathways and their approval for use in pediatric asthma, their efficacy in children who lack type 2 biomarkers is limited (7, 8). Thus, there is a huge unmet need to resolve the immunological basis of severe disease in order to inform new treatments.

T cells play a critical role in asthma pathogenesis, as exemplified by extensive data to support their contributions to type 2 inflammatory pathways of allergic asthma (9–13). However, recent work by our group and others implicates type 1 responses orchestrated by IFN-γ in promoting the pathogenesis of severe asthma, thereby deviating from the paradigm of “type 2” disease (14–20). Specifically, analysis of cells and mediators in bronchoalveolar lavage (BAL) fluid from children and adults with severe asthma has revealed a type 1 bias, including links between type 1–like T cells expressing the chemokine receptor CCR5, and worse lung function (14, 20). Respiratory viruses are likely to be instrumental in this regard, since they are the most important trigger of wheeze episodes in children, and can amplify type 1 responses in asthma, including those involving CCR5^+^ virus-specific T cells (21–26). Notably, rhinovirus (RV) accounts for up to 60% of acute episodes in severe asthma, and its detection in approximately 30% of asymptomatic children with severe disease implicates this pathogen as a key driver of airway inflammation (5, 23, 27–29).

Despite these observations, little is known about the nature of T cells that populate the lower airways of children with severe asthma, or how viral infections in early life shape this landscape. Moreover, whether corticosteroid treatment reinforces the type 1 response remains an ongoing debate (30–33). Adding to the complex picture, pathogenic processes in the pediatric airways likely occur on a backdrop of type 1 immune surveillance inherent to the mucosal barrier, as well as maturation of the immune system. These facets, along with the logistics of sampling the diseased airways in children and challenges to pinpointing rare virus-specific T cells in the lungs, have made it difficult to resolve pathogenic T cell features in severe asthma.

Here, we aimed to unravel these facets in order to define T cell–based mechanisms governing type 1 responses in the airways of children with treatment-refractory recurrent wheeze. This was done by leveraging matched BAL and blood specimens from a highly characterized cohort of children, and employing deep phenotyping and machine learning pipelines. In so doing, we resolved complex type 1–related T cell dynamics with exquisite detail, and their relationships to RV. Additionally, we revealed discrete T cell migratory pathways spanning the blood and airways that culminated in tissue residency, as well as pathways indicating transitions between discrete T cell functional populations in situ that contain RV-responsive T cells. Together, our findings spotlight the aberrant nature of type 1 immunity in the airways of children with recurrent wheeze, and the integral role of RV as a driver.

## Results

### Characteristics of study participants.

Study participants were 32 predominantly male children (71.9%), ages 1–17 years with treatment-refractory recurrent wheeze (Table 1). All had failed standard therapy and thus, underwent clinically indicated diagnostic bronchoscopy (Supplemental Figure 1; supplemental material available online with this article; https://doi.org/10.1172/jci.insight.189480DS1). All children were asymptomatic for an acute respiratory infection. Criteria for treatment failure included persistent troublesome symptoms, recurrent unscheduled health care access for wheeze, and/or persistent airflow limitation. At the time of bronchoscopy, 25 patients (78%) were on controller medications, including 24 (75%) who received daily ICS. Use of non-steroidal controllers increased with age (*P* = 0.02). Four patients received type 2 biologics (dupilumab [anti–IL-4Rα], mepolizumab [anti–IL-5], or omalizumab [anti-IgE]), and 3 were on oral prednisone. Most patients had evidence of a past or recent infection based on a history of pneumonia (67.7%) or positive viral nucleic acid PCR and/or bacterial culture from BAL fluid (53.1%). Among those with respiratory pathogens, 13 patients tested PCR-positive for viruses, and RV was most commonly detected (*n* = 10). Bacteria were detected in 10 patients, with cultures identifying *Haemophilus influenza*, *Moraxella catarrhalis*, *Pseudomonas aeruginosa*, *Staphylococcus aureus*, and *Streptococcus pyogenes*. Thus, despite being asymptomatic for infection, detection of respiratory pathogens, and RV in particular, was prevalent in children with recurrent wheeze.

### Mixed type 1 and type 17 signatures dominate the T cell landscape in the diseased airways.

**Figure 1:**
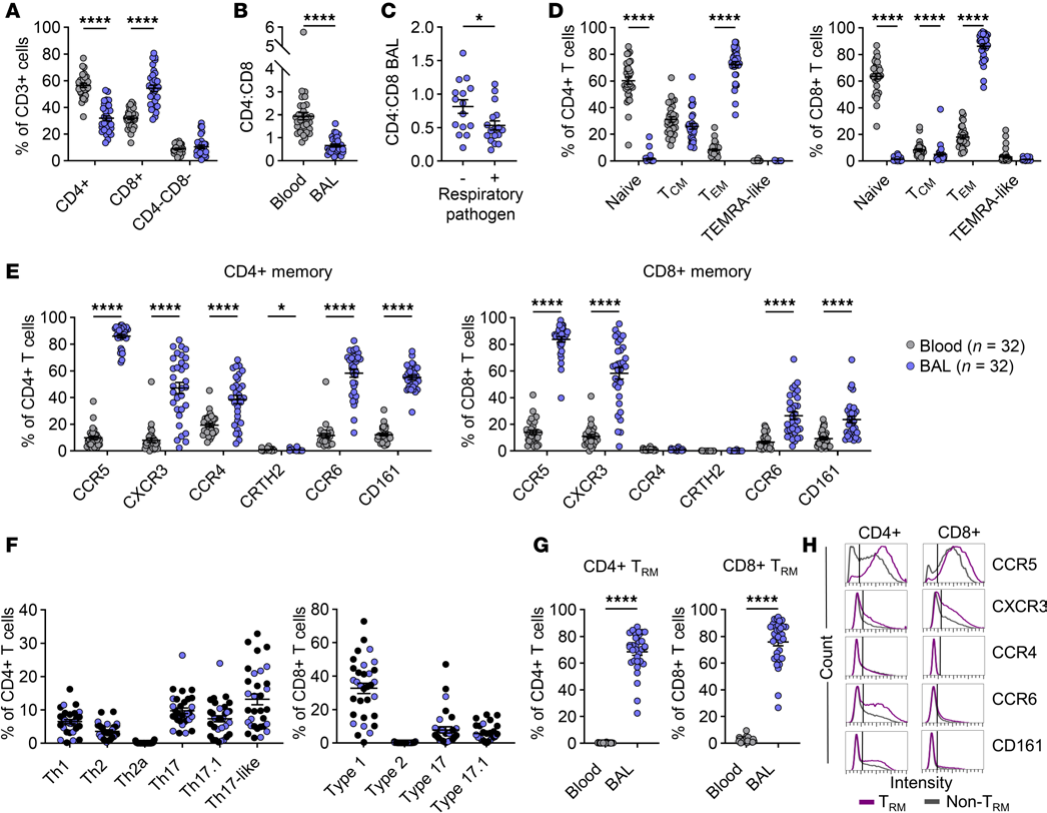
Mixtures of type 1 and type 17 signatures dominate the T cell landscape in the lower airways of children with recurrent wheeze. (**A**) T cell frequencies in matched blood and BAL, as a percentage of CD3^+^ cells. (**B**) CD4^+^/CD8^+^ T cell ratio in blood and BAL. (**C**) CD4^+^/CD8^+^ T cell ratio in the BAL of patients with (*n* = 17) or without (*n* = 15) a respiratory pathogen. (**D**) Frequencies of naive (CD45RO^–^CCR7^+^), central memory (CD45RO^+^CCR7^+^, Tcm), effector memory (CD45RO^+^CCR7^–^, Tem), and terminal effector memory–like (CD45RO^–^CCR7^–^CD27^–^, TEMRA-like) T cells in blood and BAL, as a percentage of CD4^+^ and CD8^+^ T cells. (**E**) Frequencies of marker-positive memory (Tcm, Tem, and TEMRA-like) CD4^+^ and CD8^+^ T cells in blood and BAL. (**F**) Frequencies of type 1, type 2, and type 17 subsets in BAL. Black symbols denote atopic patients (*n* = 20). (**G**) Frequencies of CD4^+^ and CD8^+^ tissue-resident memory T cells in blood and BAL (CD69^+^CD103^–^ and CD69^+^CD103^+^, Trm). (**H**) Histograms depicting the expression of select surface receptors on CD4^+^ and CD8^+^ Trm and non-Trm cells in BAL. Bars denote mean ± SEM (**A**–**G**). *P < 0.05, ****P ≤ 0.0001 by multiple Wilcoxon’s tests with Holm-Šidák correction (**A**, **D**, and **E**), Wilcoxon’s matched-pairs signed rank test (**B** and **G**), or Mann-Whitney test (**C**).

We first sought evidence for recruitment and antigen priming of type 1–like T cells in the airways of children with treatment-refractory wheeze, by comparing matched BAL and blood specimens. As expected, high-dimensional spectral flow cytometry analysis using a 28-color panel (Supplemental Table 1) revealed enrichment of CD8^+^ T cells in the lungs (34, 35), and this was most marked in patients with one or more respiratory pathogen (mean ± SEM CD4^+^/CD8^+^ T cell ratio = 0.5 ± 0.07 vs. 0.8 ± 0.1, *P* = 0.03; Figure 1, A–C). The majority of T cells in the airways displayed a memory phenotype (>90%). Both CD4^+^ and CD8^+^ effector memory T cells (Tem, CD45RO^+^CCR7^–^) were markedly enriched in BAL versus blood (72.3% ± 2.3% vs. 7.7% ± 0.7% and 86.1% ± 1.8% vs. 17.4% ± 1.4%, of CD4^+^ and CD8^+^respectively), whereas frequencies of more differentiated TEMRA-like cells (CD45RO^–^CCR7^–^CD27^–^) were low and similar at both tissue sites (Figure 1D and Supplemental Figure 2A). As we reported previously (20), most CD4^+^ and CD8^+^ T cells in the airways expressed CCR5 (86.0% ± 1.4% and 83.7% ± 2.1%, respectively), a marker notable for its role in memory responses to respiratory viruses (Figure 1E) (36, 37). Probing classical markers of type 1 (CCR5, CXCR3), type 2 (CCR4, CRTH2), and type 17 (CCR4, CCR6, CD161) T cell types indicated the accumulation of type 1 and type 17 T cells in the airways (Figure 1E). While CXCR3 was the most abundantly expressed marker on both memory CD4+ and CD8+ T cells, after CCR5, CCR6 and CD161 were expressed on higher frequencies of CD4+ T cells compared with CD8+ cells. Further resolution of airway memory CD4^+^ T cell signatures identified a predominance of type 17 canonical and non-canonical subsets, including Th17 cells (CCR6^+^CD161^+^CCR4^+^), “plastic” Th17 or Th17.1 cells (CXCR3^+^CCR6^+^CD161^+^), and Th17-like cells that lacked CCR4 (CCR6^+^CD161^+^CCR4^–^) (Figure 1F and Supplemental Figure 2B). Classical Th1 cells (CXCR3^+^ only) were less frequent, whereas classical Th2 cells (CCR4^+^ only) and pathogenic Th2 effectors (Th2a, CRTH2^+^) were the least abundant. By contrast, Th2 cells were the dominant subtype in blood (Supplemental Figure 3A). CD8^+^ T cell signatures were predominantly type 1 (CXCR3^+^), with type 17 (CCR6^+^CD161^+^) and type 17.1 (CXCR3^+^CCR6^+^CD161^+^) cells also present, albeit at lower frequencies (Figure 1F and Supplemental Figure 2C). As expected, the majority of T cells in the airways were tissue resident (68.5% ± 2.6% and 75.9% ± 2.9% for CD4^+^ and CD8^+^ T cells, respectively) based on expression of CD69 with or without CD103 (Figure 1G). Tissue-resident memory T (Trm) cells expressed higher levels of chemokine receptors as compared with their non-resident counterparts (Figure 1H). No differences in the frequencies of type 1 or 17 subsets or broader subsets were identified in the BAL according to sex (Supplemental Figure 3, B and C, and Supplemental Figure 4), whether a respiratory pathogen was present (virus and/or bacteria; Supplemental Figure 5, A–C), or on the basis of ICS dose (Supplemental Figure 5D) (38–40). Frequencies of type 1 and 17 subsets in the BAL were similar regardless of atopic status (Supplemental Figure 6A). However, the levels of total IgE among atopic patients positively correlated with frequencies of Th1 cells (*r* = 0.56, *P* = 0.01), and frequencies of Th1 cells in these patients were inversely correlated with Th17-like cells (Supplemental Figure 6, B and C). Analysis of T cells by age implied preferential accumulation of type 1–like CD4^+^ and CD8^+^ cells in the airways versus blood (Supplemental Figure 7). Among those patients for whom lung function tests were performed (*n* = 16), no relationships were identified with any T cell type (data not shown). Together, these results supported the priming and enrichment of type 1– and type 17–related signatures in the diseased airways irrespective of ICS treatment, and a paradoxical relationship to IgE.

### Aberrant T cell dynamics in the airways link to infection status.

**Figure 2:**
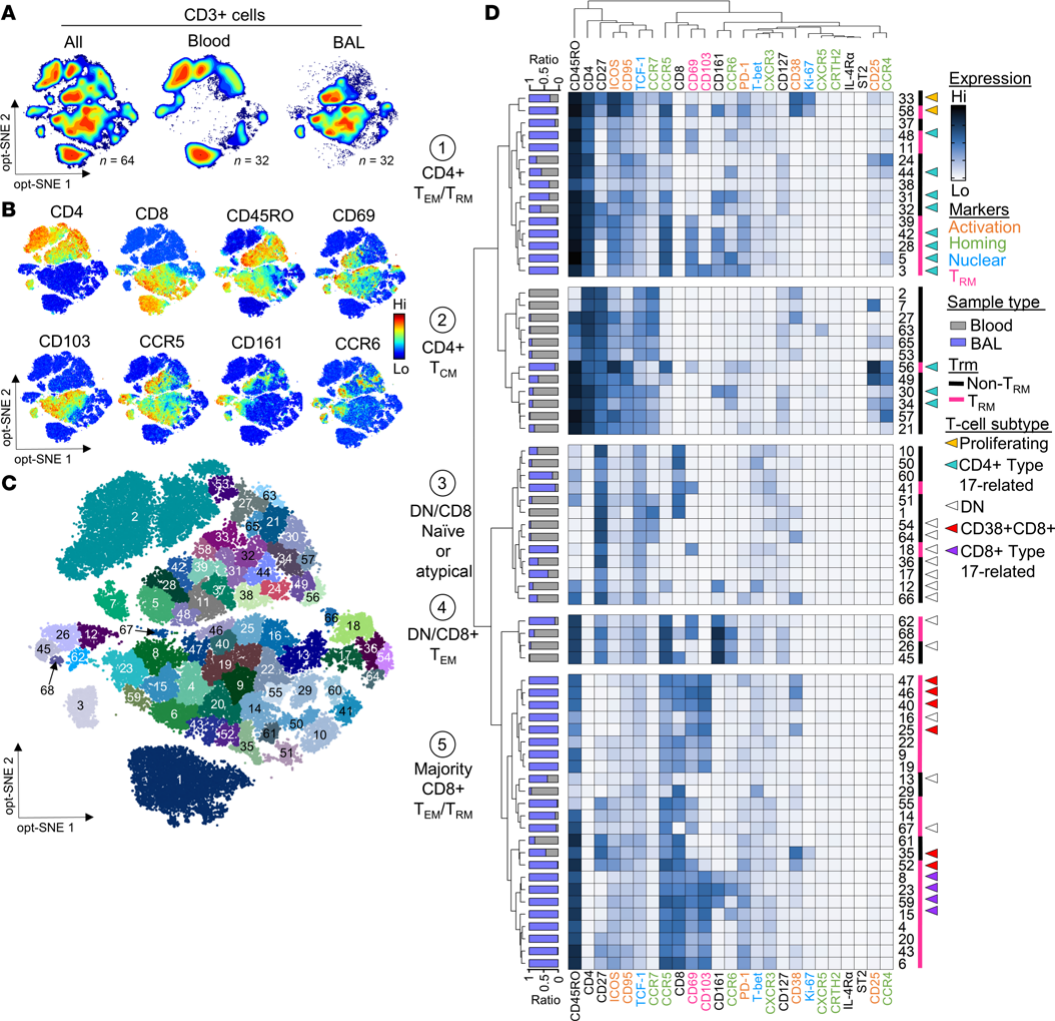
High-dimensional analysis reveals the complexity of the T cell landscape in recurrent wheeze. (**A**) opt-SNE dimensionality reduction showing the distribution of T cells in matched blood and BAL from 32 children. (**B**) Expression of select phenotypic markers across all samples (*n* = 64). (**C**) PhenoGraph clusters for all samples. (**D**) Heatmap showing the median expression of each marker according to clusters generated by PhenoGraph. Blue/gray annotation on the left denotes the proportion of cells in each cluster derived from BAL or blood.

**Figure 3:**
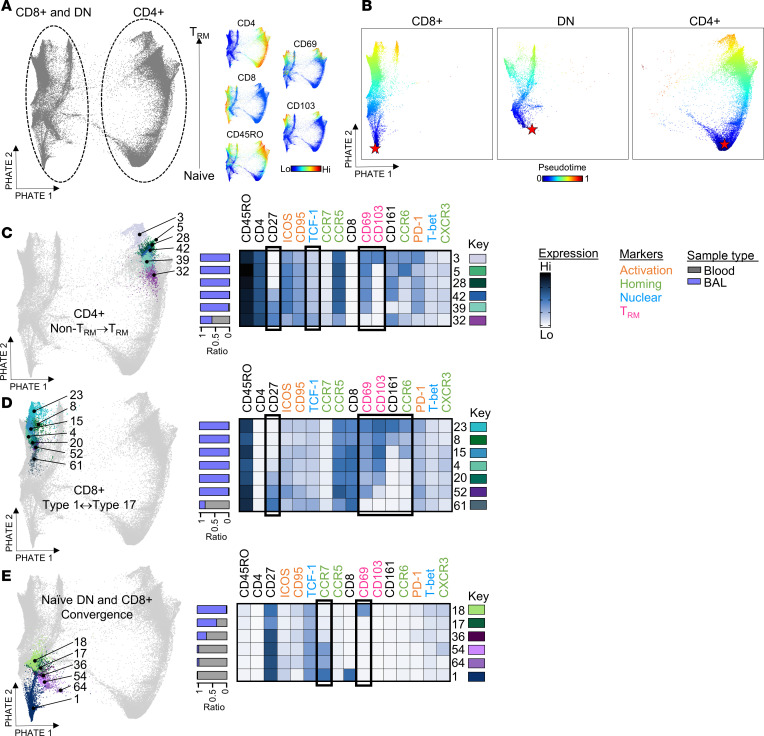
PHATE analysis captures transitions in T cell clusters in the lower airways. (**A**) PHATE map generated on CD3^+^ cells from matched blood and BAL (64 samples), with heatmaps of select markers. (**B**) Pseudotime trajectories of CD4^+^, CD8^+^, and DN T cells. The red star denotes the starting point for wishbone analysis. (**C**–**E**) PhenoGraph clusters projected on PHATE map to show transitions within CD4^+^ T cells (**C**), CD8^+^ T cells (**D**), and DN T cells (**E**). Black boxes within heatmaps contain markers that are differentially expressed between related clusters. Blue/gray annotation on the left of each heatmap denotes the proportion of cells in each cluster derived from BAL or blood.

Since we found no significant relationships between the frequencies of type 1– and type 17–related signatures in relation to infection, we next leveraged the high-dimensional nature of our data to probe the molecular complexity and dynamics of airway T cells and links to infection. Data visualization using density maps confirmed markedly different T cell distributions between airways and blood, owing to expression of CCR5 and Trm markers (CD69 and CD103) on most airway T cells and their enrichment for effector memory types (CD45RO^+^CCR7^–^) (Figure 2, A and B, and Supplemental Figure 8). Clustering analysis by PhenoGraph (41) yielded 68 distinct cell clusters across BAL and blood that reflected variable expression of a wide array of markers pertaining to lung homing, tissue residency, differentiation, activation, and effector phenotypes (Figure 2, B and C, and Supplemental Figure 8). Hierarchical clustering delineated 2 sets of cell clusters that were enriched in the airways versus the blood (groups 1 and 5) comprising mostly CD4^+^ and CD8^+^ Tem cells (CD45RO^+^CCR7^–^) that were tissue resident (CD69^+^CD103^+/–^) (Figure 2D). In contrast, cell clusters enriched in the blood (groups 2 and 3) were predominantly naive (CD45RO^–^CCR7^+^) and central memory (Tcm; CD45RO^+^CCR7^+^) cells that displayed markers expressed early in the T cell differentiation pathway (CD27 and TCF-1) (42–45). Most CD4^+^ Tem clusters in group 1 were CXCR3^lo^ Trm, and displayed variable expression of the activation markers PD-1, ICOS, and CD95 (Figure 2D and Supplemental Figure 8). Expression of the type 1–orchestrating transcription factor T-bet was low in group 1, consistent with its downregulation in tissues (46, 47). Active responses were evidenced by the presence of robustly proliferating (CD38^+^Ki-67^+^) cells (clusters 33 and 58). In addition to Th1-like clusters (CXCR3^+^T-bet^+^), memory CD4^+^ Tem unique to the airways included CD69^+^ types that were CD161^+^ or CCR6^+^CD161^+^ (clusters 3, 5, 28, 42, and 58). Low coexpression of Th1 markers by some of these subtypes indicated Th17.1 phenotypes, suggesting transitions between type 1 and type 17 functional subsets (48, 49) (Figure 2D and Supplemental Figure 8). Additional non-resident CD4^+^ Tem phenotypes were shared with those found in the blood, including Th17-like cells (CD69^–^CD103^–^CCR6^+^CD161^+^, clusters 31 and 32) that displayed increased stemness (TCF-1^+^) and were less activated (CD127^+^), perhaps reflecting recent egress from blood (50, 51). Evidence of CD4^+^ T cell transitions was supported by results of PHATE (potential of heat-diffusion affinity-based transition embedding) (52) coupled with pseudotime analysis used to model T cell progressions over time. The resulting trajectories linked cluster 32 with type 1 (cluster 39) and type 17-related Trm clusters (clusters 3, 5, 28, and 42) (Figure 3, A–C). Specifically, transitions along the path from cluster 32 (enriched in the blood) to cluster 3 (exclusive to the BAL) were associated with decreased expression of the differentiation markers CD27 and TCF-1, and a concomitant increase in tissue-resident and activation markers (CD69, CD103, and PD-1). Together, these features indicated T cell migration from the blood, establishment of tissue residency, and functional transitions in the tissue.

CD8^+^ Trm dominating the T cell landscape in the airways (group 5) displayed a predominant type 1 signature (CXCR3^+^T-bet^+^) (Figure 2D). Expression of CCR6 or CD161 was highest on 4 Trm clusters that coexpressed variable type 1 markers (clusters 8, 15, 23, and 59). PHATE analysis mapped 3 of these type 17–related Trm to the same pathway as type 1 Trm (clusters 4, 20, and 52) and non-Trm (cluster 61) signatures, further supporting transitions between type 1 and 17 CD8^+^ T cells in the lower airways (Figure 3D). Again, expression of PD-1, ICOS, and CD95 was a feature within CD8^+^ Trm, although levels and combinations varied across clusters (Figure 2D and Supplemental Figure 8). High expression of the activation marker CD38 on 6 CD8^+^ T cell clusters was notable, given that CD38 hallmarks CD8^+^ T cells that persist after viral infection (53, 54). These clusters included a subset of proliferating non-resident type 1 cells shared with blood (cluster 35) and its CD69^+^ counterpart unique to the lungs (cluster 52), as well as Trm types that lacked ICOS and PD-1 (clusters 25, 40, and 46). Interspersed among CD8^+^ Tem on the heatmap were 3 double-negative (DN, CD4^–^CD8^–^) clusters that were enriched in, or unique to, the lungs (clusters 13, 16, and 67). Also, unique to the lungs was a fourth CD69^+^ DN subset (cluster 18) that was CD45RO^–^CCR7^–^CD27^+^TCF-1^+^. This subset expressed low levels of the effector molecules CXCR3 and T-bet, and thus, did not fit with canonical naive (CD45RO^–^CCR7^+^) or effector types. Trajectory mapping showed cluster 18 at the intersection of naive DN T cells (clusters 54 and 64) and naive CD8^+^ T cells (cluster 1), pointing to convergent pathways (Figure 3E).

**Figure 4:**
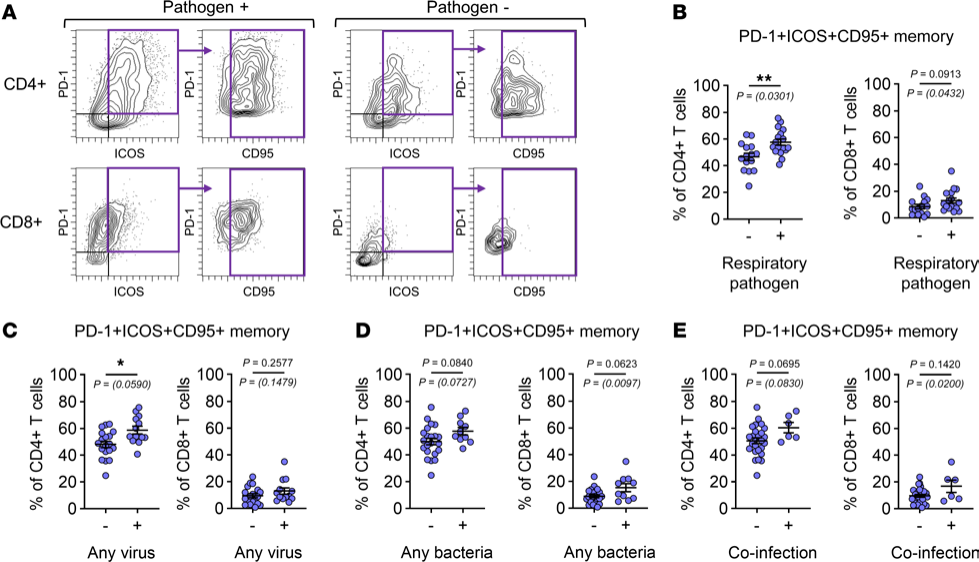
Respiratory pathogens are linked to increased T cell activation in the lower airways. (**A**) Representative scatter plots showing the gating strategy for PD-1^+^ICOS^+^CD95^+^ T cells within total memory (Tcm, Tem, TEMRA-like) T cells. (**B**–**E**) Frequencies of PD-1^+^ICOS^+^CD95^+^ memory T cells in the BAL in relation to (**B**) any respiratory pathogen (– *n* = 15, + *n* = 17), (**C**) any virus (– *n* = 19, + *n* = 13), (**D**) any bacteria (– *n* = 22, + *n* = 10), and (**E**) coinfection (– *n* = 26, + *n* = 6). Mean ± SEM. Mann-Whitney test. *P* values in parentheses are adjusted for age and sex. *P < 0.05, **P ≤ 0.01.

High expression of activation markers (PD-1, ICOS, and CD95) on Trm cells in the airway lumen indicated the presence of activated T cells in inflamed lung tissue niches. Such markers are characteristic of T cells that respond to respiratory viruses (24, 53, 55–57). Manual gating on memory CD4^+^ and CD8^+^ T cells that were PD-1^+^ICOS^+^CD95^+^ revealed higher frequencies in patients testing positive for any respiratory pathogen versus those who tested negative (Figure 4, A and B). Notably, frequencies of activated CD4^+^ T cells were significantly higher among patients for whom any viral nucleic acids were detected by PCR, whereas frequencies of both activated CD4^+^ and CD8^+^ T cells trended higher in those positive for any bacteria (Figure 4, C and D). Relationships to higher frequencies of activated T cells according to infection status were generally maintained or else strengthened after adjusting for age and sex (Figure 4, B–E, and Supplemental Figure 9). These collective findings supported aberrant T cell dynamics in the diseased airways and their link to respiratory infections.

### RV-related signatures contribute to aberrant T cell dynamics.

**Figure 5:**
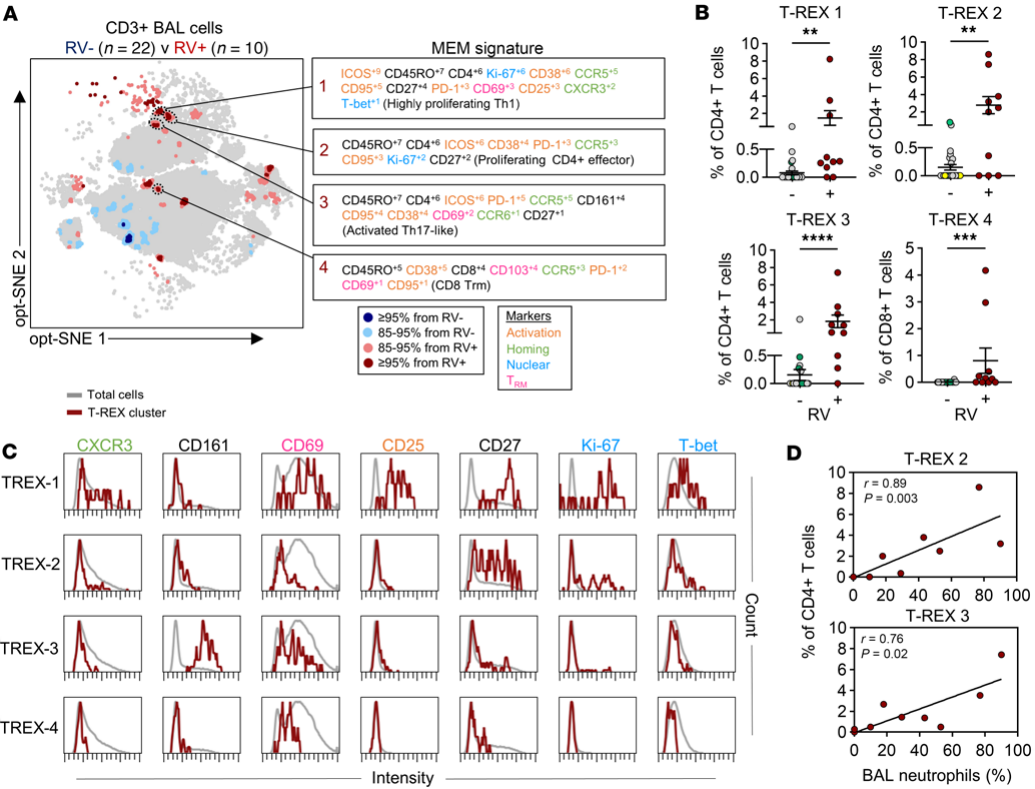
T-REX analysis reveals activated T cell signatures linked to RV infection in the lower airways. (**A**) T-REX plot comparing BAL T cells from RV^–^ (*n* = 22) and RV^+^ (*n* = 10) patients. Red populations are increased in the RV^+^ group and blue are increased in the RV^–^ group. Corresponding MEM labels are shown for 4 dominant populations increased in the RV^+^ group. Each marker in MEM labels is scored on a scale of 1-10 based on their enrichment in the population. (**B**) Frequencies of T-REX populations in RV^–^ and RV^+^ patients. Green symbols denote patients positive for other viruses (*n* = 3). Yellow symbols denote patients currently receiving a biologic (*n* = 4). Mean ± SEM. (**C**) Histograms comparing the expression of select markers on T-REX populations (*n* = 32). (**D**) Spearman’s correlations between frequencies of BAL neutrophils and T-REX populations in RV^+^ patients (*n* = 9). Lines denote linear regression. Mann-Whitney test (**B**). **P ≤ 0.01, ***P ≤ 0.001, ****P ≤ 0.0001.

**Figure 6:**
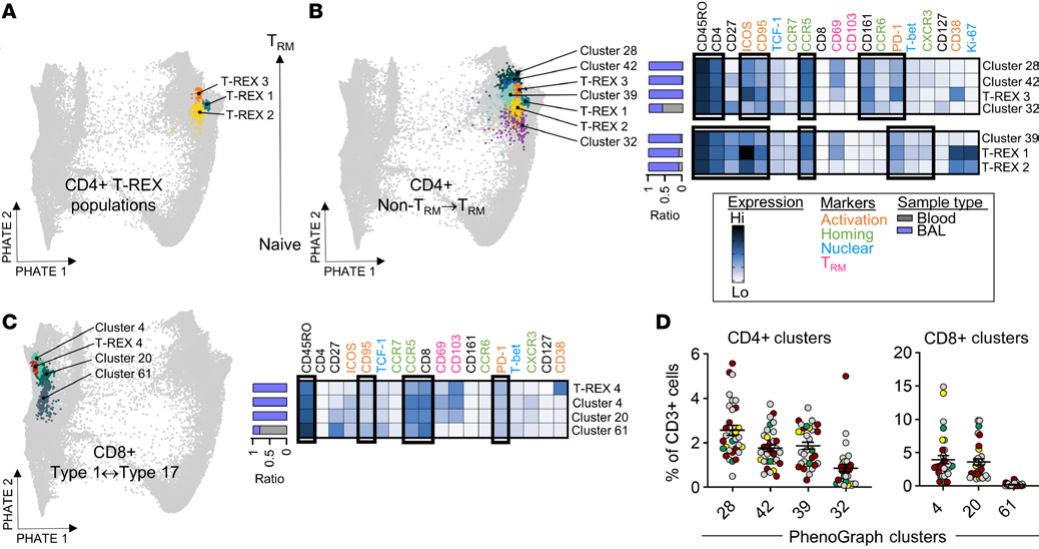
RV-related signatures map to T cell differentiation pathways in the lower airways. (**A**) Projection of T-REX populations 1–3 on a PHATE map generated on CD3^+^ cells from matched blood and BAL (64 samples). (**B** and **C**) PHATE maps with overlay of PhenoGraph clusters related to T-REX populations. Heatmaps show marker expression profiles for T-REX populations and related PhenoGraph clusters. Black squares on heatmaps contain markers expressed on all populations. Blue/gray annotation on the left of each heatmap denotes the proportion of cells in each population derived from BAL or blood. (**D**) Frequencies of PhenoGraph cluster related to T-REX 1–4 in the pediatric cohort. Colored symbols denote patients positive for other viruses (green, *n* = 3), RV^+^ patients (red, *n* = 10), patients negative for any virus (gray, *n* = 15), and patients who received a biologic (yellow, *n* = 4). Mean ± SEM.

We next asked whether RV, a major instigator and exacerbator of childhood wheeze, might act as a driver of T cell changes in the lungs. The machine learning tool T-REX (tracking responders expanding) (55) was used to pinpoint candidate virus-specific signatures, which we hypothesized would be enriched in the airways versus blood, but nonetheless present at very low frequencies. T-REX is ideally suited for this purpose, since it was designed to detect significant quantitative changes in rare T cell types displaying complex signatures, according to infection status. T cells were compared between patient groups with and without RV infection based on PCR positivity (RV^+^, *n* = 10; RV^–^, *n* = 22) (Supplemental Table 2). While demographic and clinical characteristics were similar between groups, frequencies of airway neutrophils were increased in the RV^+^ group (median 29.0% vs. 2.5%; *P* = 0.03), and bacterial coinfection was more common (median 40.0% vs. 9.1%, *P* = 0.06), similar to what has been reported (58, 59). Three subjects in the RV^–^ group tested positive for adenovirus (*n* = 1) or metapneumovirus (*n* = 2), whereas all RV^+^ patients tested negative for other viruses. T-REX analysis of airway T cells, coupled with marker enrichment modeling (MEM) (60) to assign complex molecular signature labels, identified 4 “dominant” signatures (designated T-REX 1–4) that were broadly represented and significantly enriched in the RV^+^ group versus the RV^–^ group (Figure 5, A and B). By contrast, nominal differences between groups were observed in the blood (Supplemental Figure 10A). T-REX populations 1–4 mapped to PhenoGraph clusters that were also enriched in RV^+^ children (Supplemental Figure 10B). T-REX populations 1–3 were memory CCR5^+^CD4^+^ T cells that expressed high levels of ICOS and other activation markers (CD38, PD-1, and CD95) (Figure 5A). T-REX 1 mapped to PhenoGraph cluster 33, displayed robust proliferation (Ki-67^hi^), and constituted a resident Th1 phenotype (CXCR3^+^T-bet^+^CD27^+^CD69^+^) that was CD25^+^ (Figure 5, A and C). T-REX 2, which also mapped to PhenoGraph cluster 33, was a non-resident (CD69^–^) counterpart of T-REX 1 displaying lower proliferation, consistent with a precursor that had not yet acquired tissue residency. T-REX 3 was a resident Th17-like population (CD161^+^CCR6^lo^CD69^+^) that mapped to PhenoGraph cluster 58, whereas T-REX 4 contained CD8^+^ Trm cells that were CD38^+^PD-1^+^, mapping to cluster 47. Notably, frequencies of T-REX 2 and 3 correlated with neutrophil frequencies in the airways (Figure 5D). Projection of T-REX 1–3 onto T cell trajectory pathways confirmed the relatedness of T-REX 1 and 2, and indicated increased differentiation of T-REX 3 (Figure 6A). Projecting PhenoGraph clusters onto these pathways revealed “less-activated” (CD38^–^) Trm counterparts of T-REX 1 and 2 (cluster 39) and T-REX 3 (clusters 28 and 42), as well as a blood precursor (cluster 32) (Figure 6B). PhenoGraph counterparts for the CD8^+^ population, T-REX 4, were also identified, including “less-activated” (clusters 4 and 20) and precursor (cluster 61) populations (Figure 6C). These T-REX–related clusters were present at appreciable frequencies in both RV^–^ and RV^+^ patients (Figure 6D).

Five additional “highly variable” T-REX populations (each containing >50% of cells from a single patient; T-REX 5–9) were enriched in the airways of RV^+^ patients (Supplemental Figure 11). These included a CD161^+^ memory CD4^+^ signature shared with blood that mapped to PhenoGraph cluster 32 (T-REX 5), and 3 resident CD8^+^ effector populations unique to the lungs (T-REX 6–8) that expressed variable levels of CD38, PD-1, and CD95 (mapping to clusters 19, 43, and 47). T-REX 9 corresponded to the only DN cluster that was unique to the airways (cluster 18). Analysis of the frequencies of T-REX populations 1–9 confirmed their marked enrichment in BAL versus blood in the RV^+^ group, and found no differences based on the presence of bacterial pathogens (Supplemental Figure 12). Together, these results pointed to the expansion and persistence of RV-related T cell signatures in the diseased airways, and their contribution to aberrant T cell dynamics.

### T-REX populations in the diseased airways display RV-responsive hallmarks.

**Figure 7:**
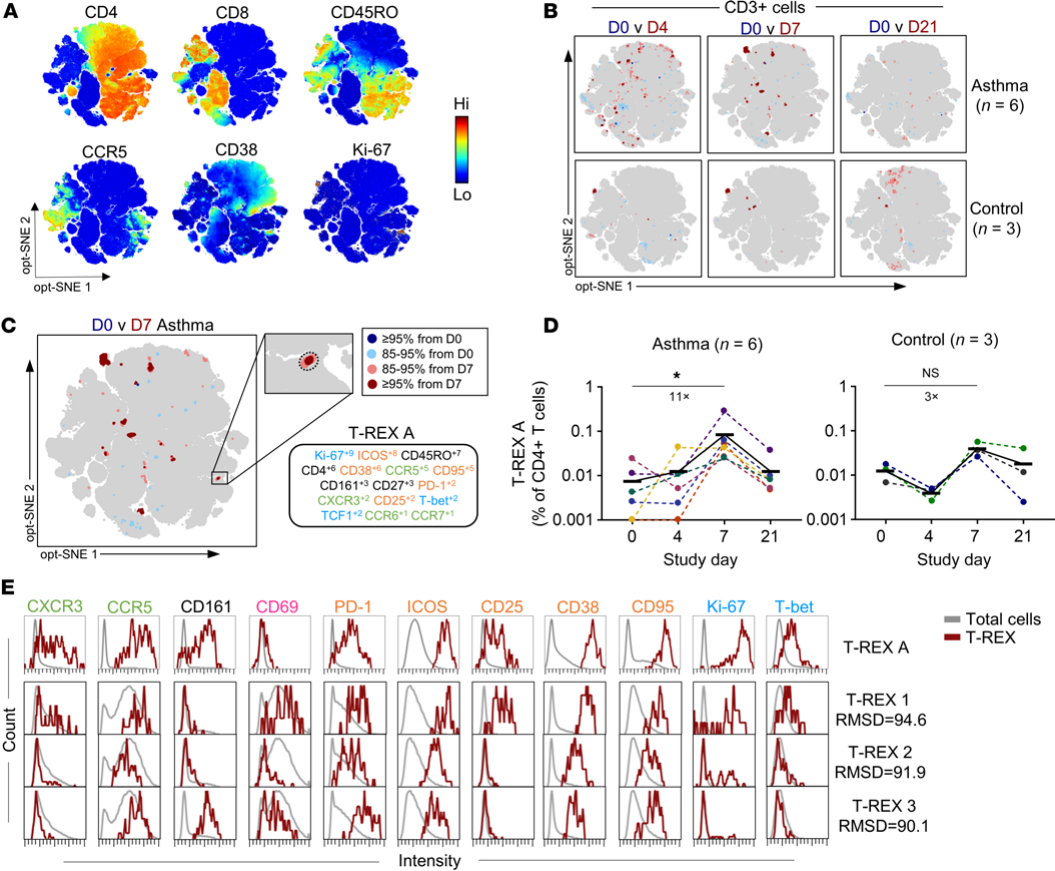
T cell signatures enriched in the lower airways of RV^+^ children display RV-responsive hallmarks. (**A**) opt-SNE maps showing expression of key markers across samples from adults with asthma (*n*=6) and healthy controls (*n*=3) during RV challenge. (**B**) T-REX plots comparing day 0 of RV challenge with days 4, 7, and 21 after challenge. (**C**) MEM signature of RV-induced T-REX A population in the blood. (**D**) Change in frequencies of T-REX A population during RV challenge, with average fold increase on day 7 versus day 0. Horizontal lines denote mean frequencies. (**E**) Histograms showing the expression of markers of interest for T-REX A (blood of RV-challenged adults, *n* = 6) and similar populations T-REX 1–3 that are enriched in the BAL of RV^+^ children (*n* = 32). Friedman’s test with Dunn’s multiple-comparison test (**D**). *P < 0.05.

To further probe whether RV-related signatures in the airways were virus responsive, we next analyzed their similarity to circulating RV-specific T cells whose expansion peaks on day 7 in the blood after RV challenge (24). We previously confirmed the ability of T-REX to detect these expanded RV-specific cells in the blood with the same precision as MHCII/peptide tetramer staining (55). Applying T-REX analysis to blood specimens from RV-challenged young adults with and without asthma revealed expansion of multiple CD4^+^ and CD8^+^ populations after infection (Figure 7, A and B, Supplemental Figure 13, and Supplemental Table 3). Among these, only one CD4^+^ Tem population (designated T-REX A) was expanded in all participants on day 7 (average 11-fold versus baseline). Its frequencies, expression of CCR5, and amplified expansion in asthma versus healthy controls were consistent with RV-specific T cells identified by tetramer staining in our prior study (24). T-REX A showed strong proliferation (Ki-67^hi^), was armed for lung homing (CCR5^+^), highly activated (ICOS^hi^CD38^hi^CD95^hi^CD25^mid^PD-1^mid^), and displayed Th17.1 features (CXCR3^+^CD161^+^CCR6^lo^T-bet^+^) (Figure 7, C and D). Using root mean square deviation (RMSD) analysis (60) to quantitatively compare the MEM label of T-REX A to signatures enriched in children’s airways revealed high similarity with T-REX 1 (proliferating Th1 Trm), T-REX 2 (proliferating CD4^+^ effector), and T-REX 3 (Th17-like Trm) (94.6%, 91.9%, and 90.1%, respectively) (Figure 7E). Thus, RV-related signatures in the diseased airways of RV^+^ children display hallmarks of RV-specific T cells, and are armed for viral activation and phenotypic transitions.

### Multifunctional and cytotoxic CD4^+^ and CD8^+^ T cells populate the diseased airways.

**Figure 8:**
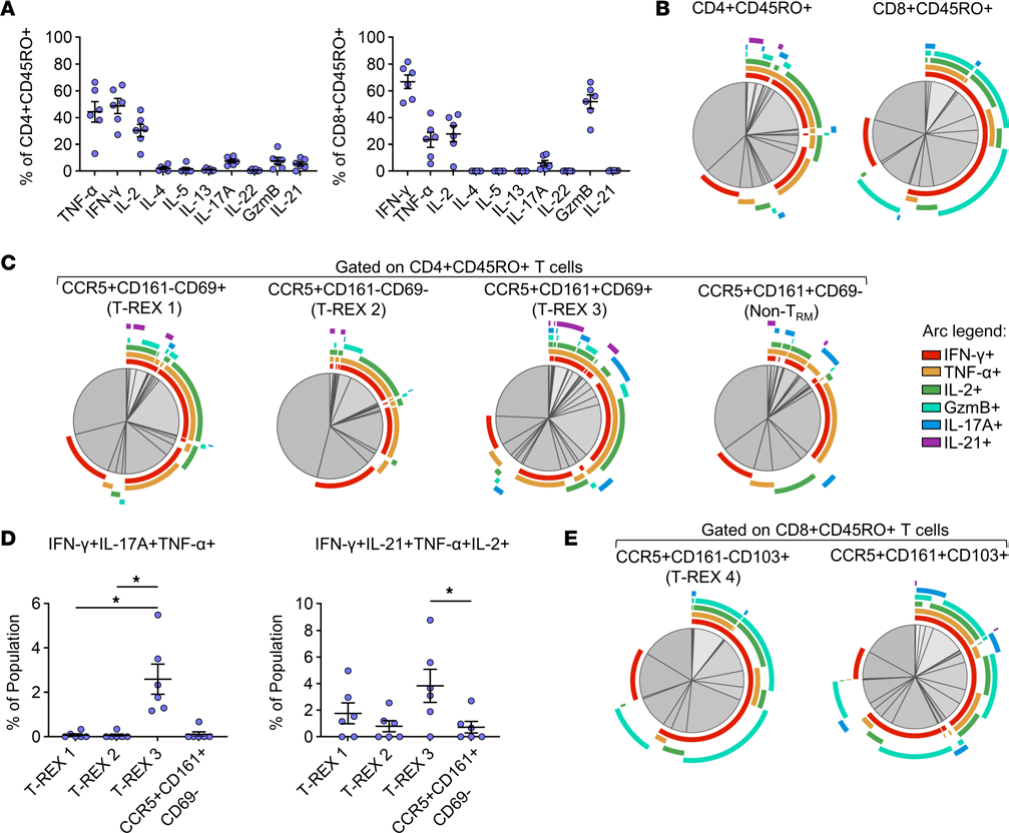
Polyfunctional T cells populate the lower airways of children with recurrent wheeze. (**A**) Frequencies of cytokine-positive cells in the BAL, as a percentage of CD4^+^ and CD8^+^ memory T cells (*n* = 6). (**B**) SPICE plots showing average cytokine signatures of CD4^+^ and CD8^+^ memory T cells in BAL. (**C**) Average cytokine signatures of CD4^+^ candidate T-REX populations. A non-Trm (CD69^–^) counterpart of T-REX 3 is shown as a comparison. (**D**) Frequencies of polyfunctional signatures within each CD4^+^ candidate T-REX population. (**E**) Average cytokine signatures of CD8^+^ T-REX 4–like signature and its CD161^+^ counterpart. Mean ± SEM (**A** and **D**). Friedman’s test with Dunn’s multiple-comparison test (**D**). *P < 0.05.

To test proinflammatory effector functions of T cells in the airways, we analyzed an array of intracellular cytokines in BAL T cells from an additional 6 children with recurrent wheeze (Supplemental Tables 4 and 5). Most cytokine-positive CD4^+^ memory T cells were IFN-γ^+^ (48.6% ± 5.6% of memory CD4^+^) and TNF-α^+^ (44.3% ± 7.7%), and around one-third were IL-2^+^ (30.4% ± 4.6%) (Figure 8A). Lower frequencies of cytotoxic (GzmB^+^) CD4^+^ (7.7% ± 2.5%), Th17 (IL-17A^+^, 7.5% ± 1.2%), and T follicular helper–like (IL-21^+^, 5.4% ± 1.5%) cells were also identified, whereas Th2-like cells (IL-4^+^, IL-5^+^, IL-13^+^) were infrequent. In contrast with CD4^+^ T cells, memory CD8^+^ T cells were primarily IFN-γ^+^ (66.8% ± 5.1%) and GzmB^+^ (51.9% ± 5.1%), whereas frequencies of TNF-α^+^, IL-2^+^, and IL-17A^+^ cells were lower (23.4% ± 5.6%; 27.8% ± 5.9%; and 6.0% ± 2.1%, respectively) (Figure 8A). Simplified presentation of incredibly complex evaluations (SPICE) analysis (61) of cytokine combinations within CD4^+^ and CD8^+^ memory T cells confirmed coexpression of IFN-γ and TNF-α in both CD4^+^ and CD8^+^ T cells, as well as triple- and quadruple-positive subsets expressing a combination of IL-2, IL-17A, IL-21, and GzmB (Figure 8B and Supplemental Figure 14A).

To analyze effector functions of virus-responsive signatures, we used a MEM-directed gating strategy based on a minimal marker set to identify populations in the cytokine assay that aligned with dominant T-REX populations 1–4 in RV^+^ children (T-REX 1: CCR5^+^CD161^–^CD69^+^, T-REX 2: CCR5^+^CD161^–^CD69^–^, T-REX 3: CCR5^+^CD161^+^CD69^+^, T-REX 4: CCR5^+^CD161^–^CD103^+^). The 4-hour stimulation period minimized changes in expression of CCR5, CD69, and CD103; however, CXCR3 was excluded from analyses, owing to its downregulation upon stimulation (Supplemental Figure 14B). Cytokine profiles of all candidate T-REX populations were dominated by IFN-γ and TNF-α. However, the CD4^+^ signature resembling T-REX 3 (CCR5^+^CD161^+^CD69^+^) displayed increased polyfunctionality (IFN-γ^+^IL-17A^+^TNF-α^+^ and IFN-γ^+^IL-21^+^TNF-α^+^IL-2^+^) compared with its CD161^–^ and non-Trm (CD69^–^) counterparts, indicating enrichment of Th17.1 and T peripheral helper-like cells within this population (Figure 8, C and D, and Supplemental Figure 14C) (62). As expected, the majority of the CD8^+^ subset resembling T-REX 4 (CCR5^+^CD161^–^CD103^+^) was GzmB^+^IFN-γ^+^ and coexpressed TNF-α or IL-2, but had lower IL-17A expression compared with its CD161^+^ counterpart (Figure 8E and Supplemental Figure 14D). These results confirmed that virus-responsive signatures in the diseased airways are equipped to exert polyfunctional and cytotoxic functions that promote type 1 and type 17 responses.

## Discussion

Although recent studies implicate type 1 responses in the pathogenesis of severe asthma in adults (14, 18, 19), the T cell landscape in the airways of children with severe recurrent wheeze remains largely unexplored. Our study used deep phenotyping methods and machine learning tools to address this major knowledge gap. Furthermore, we sought to establish a mechanistic link between a major viral trigger and T cell dynamics in the diseased airways. Rigorous investigation of T cell attributes in the context of clinical and demographic variables demonstrated T cell dynamics in the diseased airways that were dominated by complex mixtures of type 1 and type 17 CD4^+^ and CD8^+^ T cell effectors. Integral to these dynamics were migratory pathways spanning the blood and airways that culminated in tissue residency, as well as phenotypic relationships indicating transitions between type 1 and type 17 functional subsets. By using powerful machine learning methods to detect rare T cell populations, we defined RV-responsive elements that mapped to T cell trajectories. To our knowledge, our study is the first to use this approach to establish RV as a potential driver of T cell behavior in the airways. Our innovative strategy surmounted barriers to identifying virus-specific T cells, whose very low numbers and unknown epitope specificities in airway samples with limited total T cell numbers, preclude identification by labeling with MHCII/peptide tetramers. Based on our collective findings, we propose that the high incidence of viral infections, and RV in particular, contributes to a vicious cycle of T cell–based inflammation and corticosteroid resistance in susceptible children. By helping to solve the conundrum of RV as an instigator and/or driver of disease, our study informs both the immunopathology of recurrent wheeze and potential therapeutic targets to alter the clinical course.

Our results showed that starting from approximately 18 months of age, the vast majority of both CD4^+^ and CD8^+^ T cells in the airways are CCR5^+^ Trm, indicating early accumulation of memory T cells in lung tissue (63–65). While type 1–related T cells featured prominently, type 2 signatures, a hallmark of allergic asthma, were infrequent. This was borne out by expression of IFN-γ in the majority of cytokine-expressing CD4^+^ and CD8^+^ T cells. Notably, a large proportion of airway CD4^+^ T cells also displayed type 17–related features, as evidenced by their coexpression of CD161 and CCR6 with or without CXCR3 and CCR4, indicating the presence of Th17, Th17.1, and Th17-like varieties. Moreover, expression of IL-17A by both CD4^+^ and CD8^+^ T cell subsets expressing CD161 indicated contributions of bona fide type 17 cells capable of secreting IL-17 (66). Previous studies in children and adults have linked IL-17A in the serum and airways to asthma severity (67–70). In addition, CCR5^+^CD161^+^CD4^+^ T cells have been linked to decreased lung function in adult patients (14). IL-17A exerts its effects on multiple cell types, including airway smooth muscle cells, endothelial cells, and fibroblasts, and is arguably best known in asthma for mediating the recruitment of neutrophils to the lungs (71–76). The interplay between type 17 inflammation and corticosteroids, as well as insensitivity of Th17 cells to corticosteroid-mediated cell death, may serve to compound type 17–mediated lung inflammation in severe asthma (40, 77–79).

In addition to CD4^+^ and CD8^+^ T cell types, our study identified DN T cells (CD4^–^CD8^–^) in the airways, including an atypical tissue-resident type 1 signature (cluster 18) that was unique to this site. DN T cells in the lungs, which may include γδ T cells (reported to be ~13% of total CD3^+^ cells in the lungs of children), respond rapidly to respiratory viruses, and can exert regulatory and effector functions (80, 81). As such, these cells warrant further investigation, including their ties to the production of autoantibodies, which have been linked to asthma exacerbations and severe disease (82–85).

Our results revealed a highly dynamic T cell landscape in the diseased airways as evidenced by a broad spectrum of signatures of activation, proliferation, and tissue residency across multiple related populations. In particular, coexpression of PD-1, ICOS, and CD95 pointed to a high degree of T cell activation. This was observed in RV-responsive T cell signatures undergoing robust proliferation, congruent with RV-specific T cell signatures identified during our prior studies of experimental RV infection (24, 55, 86, 87). Coexpression of activation markers was also observed on broader T cell populations displaying complex type 1 and type 17 signatures. In particular, the presence of Th17.1 cells indicated active transitions between type 1 and type 17 cells, a finding bolstered by trajectory mapping, and detection of T cell populations coexpressing IL-17 and IFN-γ. These data, coupled with migratory pathways spanning blood and tissue, supported a model wherein egress of T cells from the blood culminates in establishment of tissue residency, activation, and differentiation along a type 1/type 17 axis. Pinpointing Th17.1 cells in our study was important since these constitute a polyfunctional and pathogenic subset involved in diverse chronic inflammatory disorders that is also resistant to suppression by regulatory T cells in the inflamed patient (48, 88, 89). In extension of this notion, the inflamed milieu may also serve to override pathways acting to regulate the functions of lung-resident T cells through PD-1, ICOS, and CD95, thereby further amplifying the inflammatory cascade (90–93).

Our study does not exclude a role for T cell exhaustion in perpetuating airway inflammation. This could arise through chronic antigen stimulation and consequent dysregulated responses. Arguing against this, our work identified only low numbers of canonical TEMRA-like cells (CD45RO^–^CCR7^–^CD27^–^), confirming that T cells that are terminally differentiated and predisposed to cell senescence are infrequent. Moreover, the array of polyfunctional and cytotoxic signatures identified in the diseased airways indicated that T cells remained functionally competent.

The drivers of T cell dynamics in the lungs are likely to be multifactorial, owing to the complex interplay between environment and host. Factors may include age-related maturation of the immune system, exposures to respiratory pathogens, iatrogenic effects, and other susceptibility determinants that dictate the inflamed milieu (14, 64, 65, 94–97). Results of our study favor a key role for respiratory pathogens on a backdrop of immune maturation in instigating disease; these include enhanced activation of airway T cells in children who tested positive for respiratory pathogens, as well as the selective accumulation of type 1–related T cell signatures in the airways with age. Detection of RV-related T cell signatures in the airways advances this notion. Given that all RV^+^ children were asymptomatic for the common cold at the time of bronchoscopy, our data also imply that RV-responsive populations can remain expanded after acute infection, and are poised to exert potent effector functions. Persistence of such cells might be prolonged, as evidenced by our ability to detect RV-related T-REX populations in the airways of RV^–^ children, albeit at lower frequencies than in RV^+^ children, and the presence of PhenoGraph clusters containing RV-related T-REX populations in both RV^–^ and RV^+^ children. These data bolster our previous report that implicated RV-specific Th1 cells in promoting airway inflammation in adults with asthma, even in the absence of infection (24). When considering host susceptibility as a driver of T cell dynamics, our seemingly paradoxical data showing a positive correlation of serum IgE levels with frequencies of Th1 cells in the airways also fits with magnified RV-specific Th1 cells observed in adults with allergic asthma (24). This relationship is notable, since it provides a plausible T cell–based mechanism wherein the interaction between IgE and RV that is known to predispose to increased risk of acute wheeze in atopic children does so by fostering aberrant type 1 responses (22, 26). The inflamed milieu in the atopic child may also feed into T cell plasticity and shifts in functional T cell subsets, as evidenced by the inverse relationship between Th1 and Th17-like cells found in atopic children in our study.

Treatment of severe recurrent wheeze remains a major challenge. Current type 2 biologics often fail to provide symptom relief in patients with type 1 asthma. Unfortunately, biologics targeting IL-17A and its receptor have also not proven efficacious for moderate-to-severe asthma (ClinicalTrials.gov NCT01478360 and NCT03299686; refs. 98, 99). The complexity of T cell types and their associated cytokines reported here argue for the utility of combination therapy along with intervention in early life. Alternatively, identifying a single molecule that enables broad targeting of T cells might be a useful strategy. CCR5 is one such example supported by our data, whose inhibition in a mouse model of type 1 asthma has shown promise (15). That study used the CCR5 antagonist maraviroc, a drug currently approved to treat HIV. This drug also has the advantage of conserving CCR5^+^ Trm cells that are essential to barrier immunity, while preventing egress of T cells into the lungs from the periphery (100).

Our study had several limitations. Firstly, the limited numbers of total T cells in airway specimens (average of 1,200 T cells) precluded the use of tetramers to verify virus-specific signatures. Detection of rare virus-specific CD4^+^ T cells in the airways using MHCII/peptide tetramers is technically constrained by the considerations of HLA restriction and limited epitope selection (86). Antigen stimulation assays, such as activation-induced marker assays, provide an alternative method for identifying virus-specific signatures, including their cytokine profiles, and are not constrained by epitope considerations; however, signals are very unreliable where T cell numbers are limiting, phenotypes change upon T cell activation, and friable cells isolated from a chronically inflamed milieu undergo activation-induced cell death (101, 102). However, these limitations also spotlight the strengths of our approach. Specifically, the T-REX tool, which was originally developed to precisely identify rare RV-specific T cells in the RV challenge model (55), does not require the use of antigen-specific reagents, and captures the entire spectrum of virus-responsive T cells in an unsupervised fashion. This aspect, and its ability to overcome limitations in T cell numbers using combined high-dimensional T cell datasets across all patients, enables the reliable and reproducible detection of virus-related signatures where cell numbers are very limited. Another limitation was that we did not verify T cell signatures in the tissues using endobronchial biopsies, owing to restrictions on sample procurement in children. However, recent work in donors indicates that T cells present in the airway lumen are representative of those in the tissues (63). We also did not include age-matched healthy controls for ethical reasons. Thus, it was not possible to delineate T cell components of barrier immunity from pathogenic T cell types in our datasets. Nonetheless, aberrant T cell responses in the diseased airways likely include changes in protective T cell types or else their progeny. Moreover, we cannot exclude the contributions of non-RV T cell types, especially given the high viral carriage reported in type 1 high airway disease (30, 58, 103). However, our findings are substantiated by studies that compared airway T cells in adults with severe versus milder asthma, and by T cell signatures reported in prior RV challenge studies (14, 17, 19, 24, 55). Lastly, we cannot discount the influence of corticosteroids on biasing toward type 1 signatures in the airways. Arguing against this, we observed similar frequencies of both type 1 and type 17 signatures in children with and without current ICS use. Several studies have implicated corticosteroid-mediated preferential suppression of Th2 cells in favoring a type 1 bias (30, 32, 33); whereas other conflicting data in animal models points to a role for Th1 cells in mediating corticosteroid resistance (104).

In summary, our results demonstrate aberrant T cell dynamics in the airways of children with severe recurrent wheeze. These are dominated by a complex interplay between type 1 and type 17 populations, and driven by RV-responsive elements. Our work provides the rationale for T cell–based interventions in recurrent wheeze in order to mitigate the vicious cycle of inflammation and corticosteroid insensitivity underlying this debilitating disease.

## Methods

### Sex as a biological variable

Our study examined both male (72%) and female (28%) patients. Sex was used as a variable when analyzing T cell frequencies, with statistical corrections applied where appropriate.

### Study participants

#### Pediatric.

Thirty-eight children (32 for T cell phenotyping and 6 for intracellular cytokine analysis) with recurrent wheeze who underwent clinically indicated diagnostic bronchoscopies participated in the study (Supplemental Figure 1). Children with treatment-refractory symptoms, recurrent unscheduled health care access for wheeze, and/or persistent airflow limitation were considered for bronchoscopy. Not all children were on ICS at the time of bronchoscopy predominately due to the inability of the family to afford the medication, or the failure of governmental health insurance providers to approve the medication. ICS doses are based on age, with children above the following classified as high dose and those below classified as low dose: less than 6 years old, ≥176 μg/day; 6–11 years old, 500 μg/day; 12 or more years old, 1000 μg/day. Demographic and clinical information was obtained by questionnaire and review of the medical records. Additional information on the bronchoscopy and clinical tests can be found in published reports (27, 103).

#### Adult.

Samples were analyzed from 9 adults (age 19–26) who previously participated in a study of experimental RV-A16 infection (24, 105). This included participants with mild allergic asthma and healthy non-allergic controls. Participants who had confounding infections or remained uninfected were excluded from analysis. Peripheral blood specimens were obtained before (day 0) and during (days 4, 7, and 21) RV infection.

### Detection of respiratory microbes

BAL fluid was sent to the University of Virginia Medical Laboratories for bacterial cultures and multiplex PCR for respiratory pathogens, including adenovirus, coronavirus, human metapneumovirus, RV/enterovirus, influenza A and B, parainfluenza 1, 2, 3, and 4, respiratory syncytial virus A and B, *Mycoplasma* *pneumoniae*, and *Chlamydia* *pneumoniae*.

### Sample processing

PBMCs were isolated by density centrifugation using SepMate (Stemcell Technologies) and cryopreserved in FBS (Gibco) with 10% DMSO (Sigma-Aldrich). Cells were isolated from BAL fluid by way of centrifugation and cryopreserved in FBS with RPMI (Gibco) and 10% DMSO.

### T cell phenotyping by spectral flow cytometry

Cells isolated from the blood and BAL of 32 children with recurrent wheeze were analyzed for cell-surface and intracellular markers using a 28-color flow panel (Supplemental Table 1). Blood and BAL samples were run in 3 batches, each containing a standard batch control sample for normalization, with matched samples being run in the same batch. Cells isolated from the blood of adults who underwent RV challenge were also analyzed using the 28-color flow panel. In brief, cells were incubated with the viability dye LIVE/DEAD Fixable Blue (Invitrogen/Thermo Fisher Scientific) for 15 minutes at room temperature in the dark. For detection of surface markers, cells were then washed and incubated for 40 minutes at room temperature with a 100 μL mixture of antibodies, Human TruStain FcX (BioLegend), Brilliant Stain Buffer Plus (BD Bioscience), and FACS buffer (PBS, 0.5% bovine serum albumin, 0.1 M EDTA). Cells were then washed and fixed/permeabilized (eBioscience Foxp3/Transcription Factor Staining Buffer Set, Thermo Fisher Scientific) according to the manufacturer’s protocol. For intracellular markers, cells were incubated with a 100 μL mixture of antibodies and permeabilization buffer for 40 minutes at room temperature. Cells were washed, resuspended in FACS buffer, and analyzed on a 5-laser Cytek Aurora.

### Spectral flow cytometry analysis

Following acquisition, data quality control and preprocessing were performed on all samples. This included (a) spectral unmixing with autofluorescence extraction; (b) compensation; (c) manual gating to exclude doublets, dead cells, and fluorochrome aggregates; and (d) gating on the population of interest, CD3^+^ cells (Supplemental Figure 15). BAL samples with less than 50% viability in the lymphocyte gate and/or fewer than 1,000 live CD3^+^ cells were excluded from further analysis, along with their matching PBMC sample. Samples from the pediatric cohort were run in 3 batches, and to remove batch variability the normalization algorithm, CytoNorm (106), was applied to CD3^+^ cells in each sample. Normalization was validated through comparisons of UMAPs (107) generated before and after normalization using batch control samples, as well as comparisons of histograms for each normalized marker across the batch controls (Supplemental Figure 16). Following data preprocessing, optimized t-distributed stochastic neighbor embedding (opt-SNE) maps were generated by downsampling and pooling (a) matched blood and BAL samples or (b) all time points in the RV challenge for both asthmatics and controls. Intracellular markers (Ki-67, T-bet, TCF-1) were excluded in the generation of each map. To characterize the T cell landscape in the pediatric cohort, PhenoGraph clustering was performed using the opt-SNE axes and a *k* value of 30. A *k* value of 30 was chosen based on (a) testing *k* values from 16–60 to identify a range of values where cluster formation was similar between *k* values, and (b) selecting a *k* within this range that maintained resolution of subpopulations within the opt-SNE map. Phenograph clusters were paired down using RMSD (https://github.com/cytolab/cytoMEM), wherein clusters with greater than 98% similarity were grouped. To identify populations linked to RV positivity in the BAL of the pediatric cohort, the previously generated opt-SNE map was analyzed by T-REX (https://github.com/cytolab/T-REX) in R (v4.4.0; Rstudio, 2024.04.2+764). To identify T cells responding to RV infection in the challenge model, T-REX analyses were done comparing days 4, 7, or 21 of infection to day 0, separating those with asthma and healthy controls. To obtain cell frequencies for populations identified by the comparisons, T-REX files were imported into OMIQ (108), and gates were generated around populations of interest. Visualization of transitions between cell populations and inference of trajectory were done using PHATE (*k* = 20) and wishbone, on PHATE axes, in OMIQ.

### Intracellular cytokine analysis

Cells isolated from the BAL of 6 children with recurrent wheeze were analyzed for surface markers and intracellular cytokines using a 21-color spectral flow cytometry panel (Supplemental Table 5). In brief, cells were plated in a 96-well plate and stimulated with cell activation cocktail with Brefeldin A (PMA/ionomycin, BioLegend) for 4 hours at 37°C. Following incubation, cells were harvested and incubated with the viability dye LIVE/DEAD Fixable Blue (Invitrogen/Thermo Fisher Scientific) for 15 minutes at room temperature in the dark. For detection of surface markers, cells were then washed and incubated for 40 minutes at room temperature with a 100 μL mixture of antibodies, Human TruStain FcX, Brilliant Stain Buffer Plus, and FACS buffer. Cells were then washed and fixed with BD Cytofix/Cytoperm Fixation/Permeabilization Kit, according to the manufacturer’s protocol. For detection of intracellular cytokines, cells were permeabilized with the Cytofix/Cytoperm kit and incubated for 30 minutes at room temperature with a 100 μL mixture of antibodies, Brilliant Stain Buffer Plus, and BD Perm/Wash. Cells were then washed with BD Perm/Wash, resuspended in FACS buffer, and analyzed on a 5-laser Cytek Aurora. Following data acquisition, quality check and preprocessing was performed as described above. Only samples with a viability of 40% or greater were included in subsequent manual gating and high-dimensional analysis. Analysis and visualization of cytokine-positive cells within T cell subsets were done using SPICE with a 1% threshold.

### Statistics

Statistical analysis was performed using GraphPad Prism version 10.2.3. Differences in cell frequencies of paired data were analyzed using Wilcoxon’s matched-pairs signed rank test or multiple Wilcoxon’s test with Holm-Šidák correction for multiple comparisons. In contrast, Mann-Whitney test, multiple Mann-Whitney with Holm-Šidák correction, or Kruskal-Wallis test with Dunn’s correction for multiple comparisons were used for unpaired data. Within-group comparisons over time were done using Friedman’s test with Dunn’s correction for multiple comparisons. Associations between T cell subset frequencies, total IgE levels, and age were determined using Spearman’s correlations. Partial correlations were used when correcting for age. Multiple linear regressions were used when correcting for both age and sex. Between-group differences comparing patient characteristics were done using Kruskal-Wallis or Mann-Whitney test for continuous variables and Fisher’s exact test for categorical variables. The heatmap for PhenoGraph clusters was generated using median fluorescence intensity exported from OMIQ, and the R package ComplexHeatmap in R version 4.4.0. A *P* value of less than 0.05 was considered significant.

### Study approval

Studies in children were approved by the University of Virginia Human Investigations Committee (Protocols 15662 and 22487). Studies in adults were approved by the University of Virginia Human Investigations Committee and the National Institute of Allergy and Infectious Diseases Safety Committee (Protocol 12673; ClinicalTrials.gov NCT02111772). Written informed consent was obtained from all adult study participants. Legal guardians of pediatric participants provided written informed consent, and assent was obtained from older children when indicated.

### Data availability

Values for data points shown in graphs are reported in the Supporting Data Values file. Flow cytometry and deidentified human participant data are available upon request from the corresponding author. Rmarkdown for the T-REX and RMSD algorithms is available on the CytoLab Github page (https://github.com/cytolab/T-REX and https://github.com/cytoMEM).

## Author contributions

All authors conceptualized the study. NB, LMM, and JAW devised methodology. NB performed the experiments and formal analysis. KW, WGT, and JAW provided resources. NB and JAW wrote the original draft of the manuscript, which was reviewed and edited by all authors.

## Supplementary Material

Supplemental data

Supporting data values

## Figures and Tables

**Table 1:**
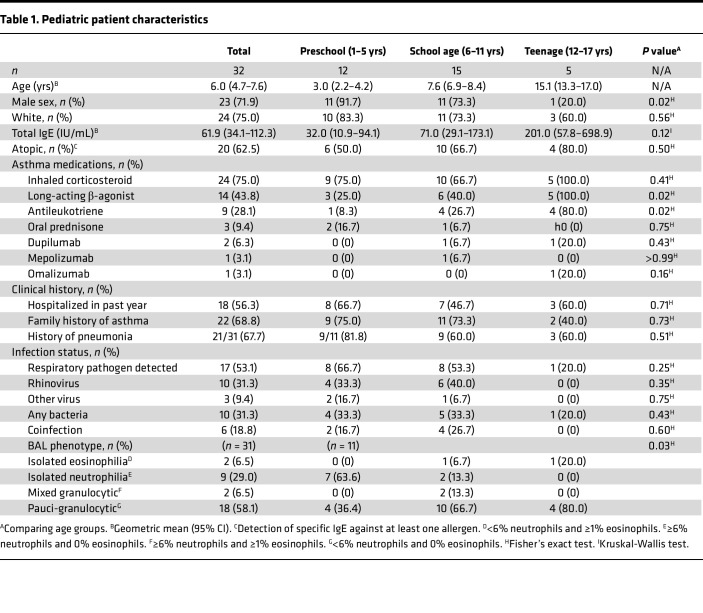
Pediatric patient characteristics

## Version 1 05/08/2025

Electronic publication
