# Supplementary material for: Rhinovirus as a driver of airway T cell dynamics in children with treatment-refractory recurrent wheeze
Source: JCI Insight. 2025 May 8;10(9):e189480. doi: 10.1172/jci.insight.189480 (PMC12128989; doi:10.1172/jci.insight.189480)
Supplement: Supplemental data [file jciinsight-10-189480-s225.pdf]

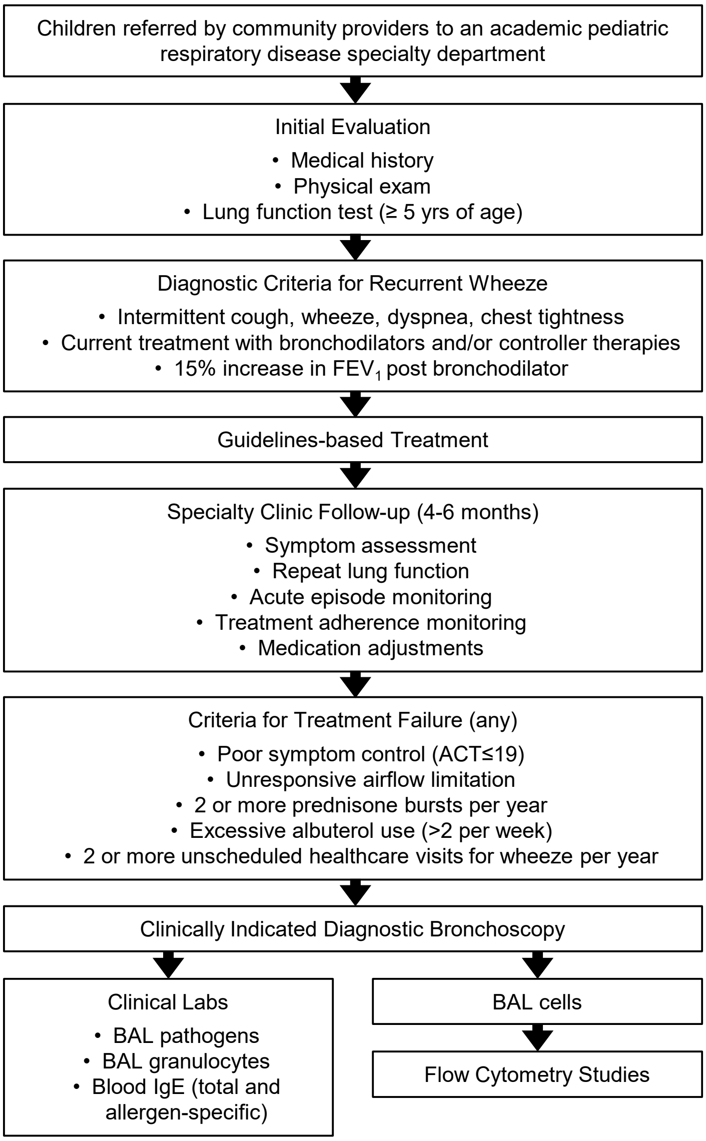

Supplemental Figure 1. Flow chart showing the clinical assessment, treatment, and indications for a clinically indicated bronchoscopy in children with treatment-refractory recurrent wheeze.

Supplemental Table 1. Spectral flow cytometry panel for T-cell phenotyping.

Marker	Color	Clone	Vendor	Catalog number	Dilution
Viability	Live/Dead Blue		Invitrogen	L23105	1:4000
CD3	BUV395	SK7	BD	564001	1:50
CD4	BUV496	OKT4	BD	750980	1:50
CD45RO	BUV563	UCHL1	BD	748369	1:100
PD-1	BUV615	EH12.1	BD	612991	1:50
CXCR3	BUV661	1C6/CXCR3	BD	741649	1:50
CCR5	BUV737	2D7/CCR5	BD	612808	1:25
CD69	BUV805	FN50	BD	748763	1:100
IL-4Rα	BV421	G077F6	Biolegend	355014	1:50
CRTH2	BV480	BM16	BD	746388	1:200
CCR4	BV605	L291H4	Biolegend	359418	1:25
CCR7	BV650	G043H7	Biolegend	353234	1:20
CCR6	BV711	G034E3	Biolegend	353436	1:50
CXCR5	BV750	J252D4	Biolegend	356942	1:50
CD161	BV785	HP-3G10	Biolegend	339930	1:20
ICOS	BB515	C398.4A	BD	565881	1:100
CD14	FITC	M5E2	Biolegend	301804	1:20
CD19	FITC	HIB19	Biolegend	302206	1:20
CD8	PerCP	SK1	Biolegend	344708	1:200
CD95	BB700	DX2	BD	566543	1:100
CD103	Per-CP eFluor710	Ber-ACT8	Invitrogen	46103742	1:50
ST2	PE	Polyclonal	R&D Systems	FAB5231P100	1:10
CD25	PE-FIRE700	M-A251	Biolegend	356146	1:50
T-bet	PE-CY7	4B10	Biolegend	644823	1:800
Ki-67	APC	20Raj1	Invitrogen	17-5699-41	1:200
TCF-1	AF647	C63D9	Cell Signaling Technology	6709S	1:200
CD127	APC-R700	HIL-7R-M21	BD	565185	1:200
CD27	APC-CY7	O323	Biolegend	302816	1:50
CD38	APC-FIRE810	HIT2	Biolegend	303550	1:50

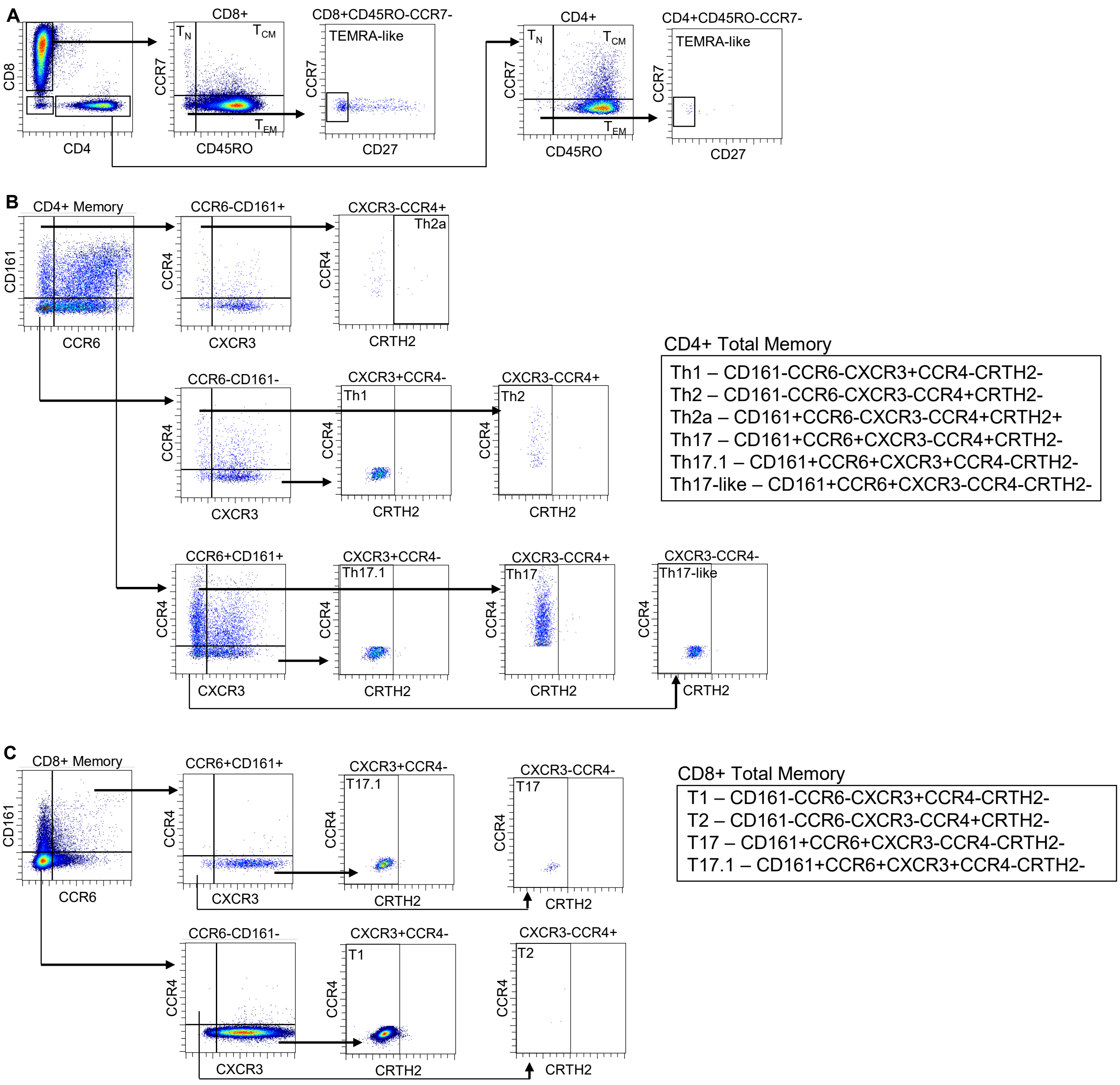

Supplemental Figure 2. Representative scatter plots showing the gating strategy for (**A**) memory T cells and (**B** and **C**) type 1, type 2, and type 17 subsets.

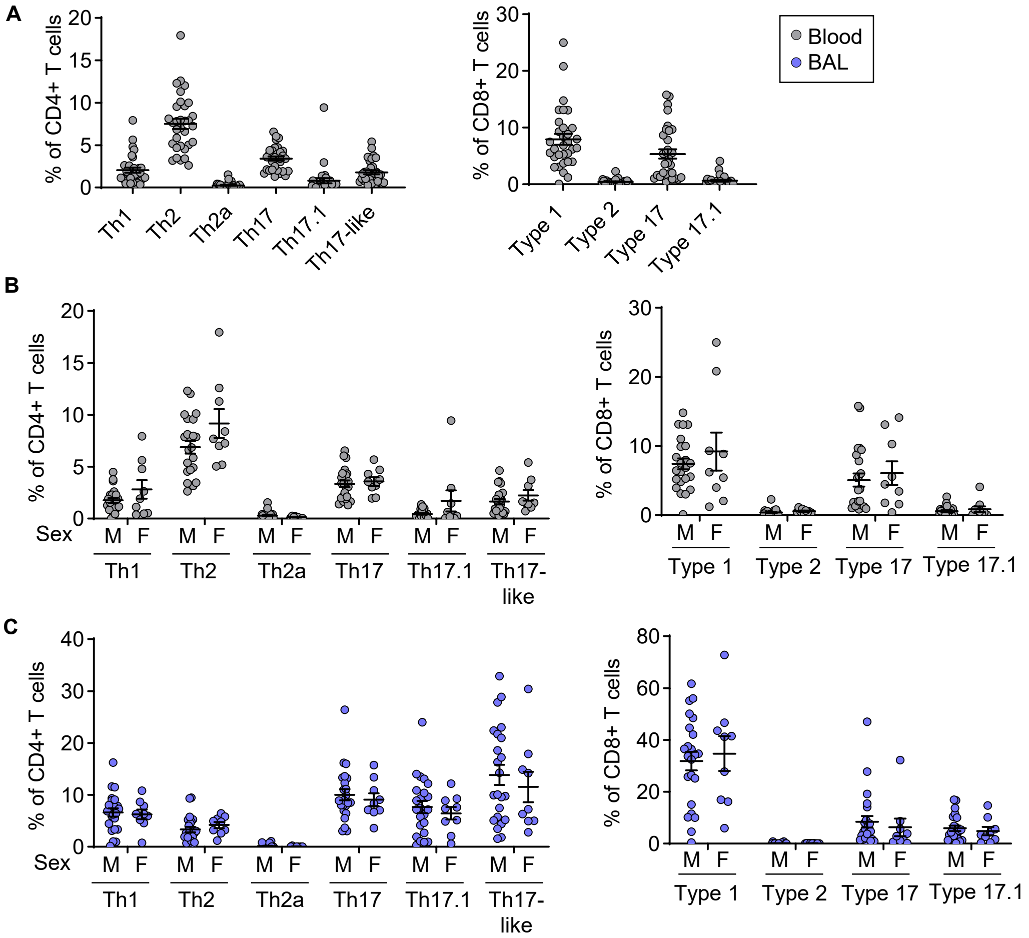

Supplemental Figure 3. Frequencies of type 1, type 2, and type 17 signatures by sex. (**A**) Frequencies of T-cell subsets in the blood (n=32). (**B** and **C**) Frequencies of T-cell subsets in the blood (**B**) and BAL (**C**) of males (n=23; M) and females (n=9; F). Bars denote mean ± SEM. Multiple Mann-Whitney tests with Holm-Sidak correction (**B** and **C**).

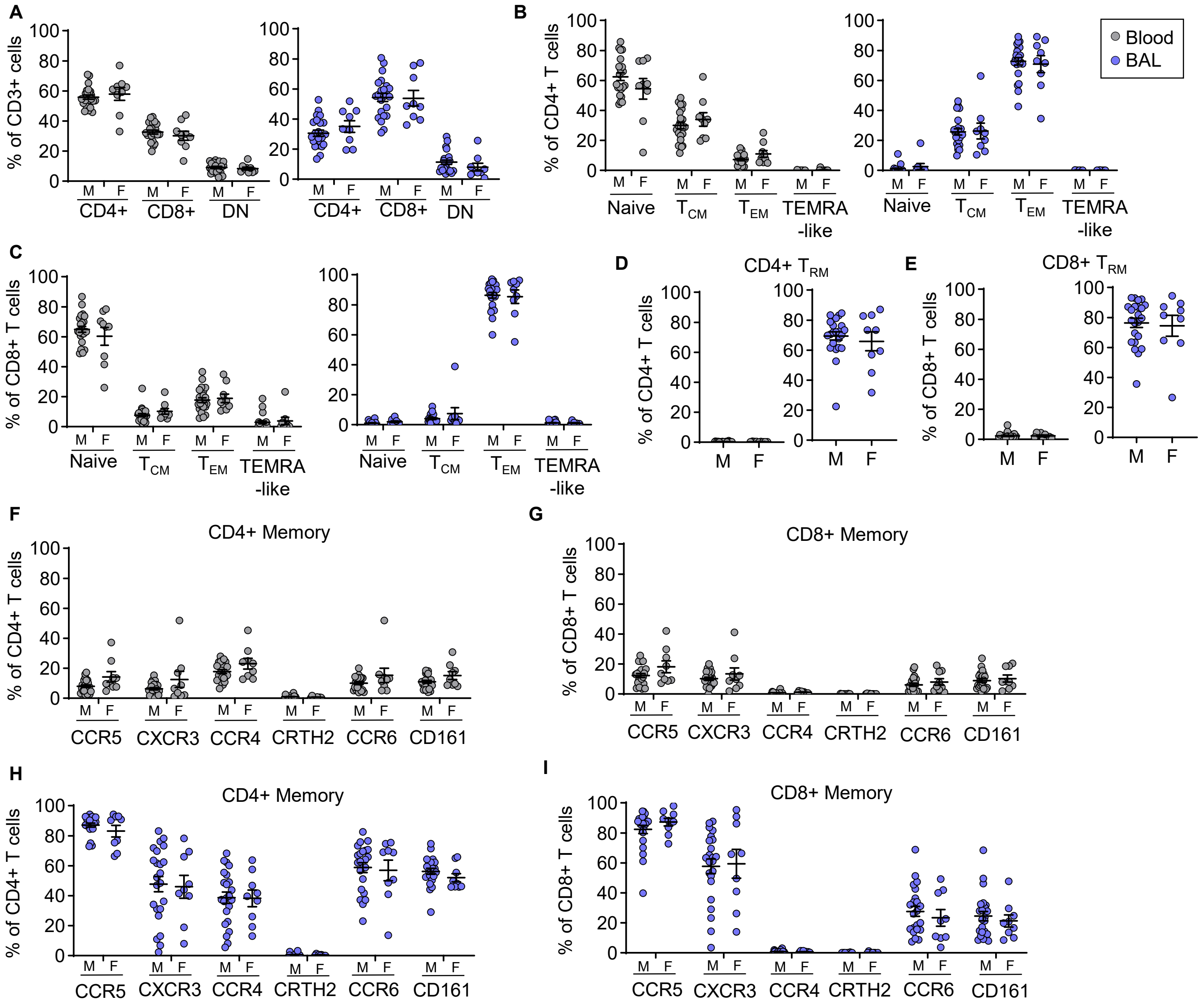

Supplemental Figure 4. Frequencies of broad T-cell subsets by sex. (**A**) Frequencies of CD4+, CD8+, and DN T cells in blood and BAL, as a percentage of CD3+ cells. (**B** and **C**) Frequencies of naïve, T_{CM}, T_{EM}, and TEMRA-like T cells in blood and BAL, as a percentage of CD4+ and CD8+ T cells. (**D** and **E**) Frequencies of CD4+ and CD8+ T_{RM} in blood and BAL. (**F-I**) Frequencies of marker-positive memory (T_{CM}, T_{EM}, and TEMRA-like) CD4+ and CD8+ T cells in blood and BAL. Mean ± SEM. Multiple Mann-Whitney tests with Holm-Sidak correction (**A, B, C, F, G, H,** and **I**) and Mann-Whitney test (**D** and **E**). M, Male, n=23; F, Female, n=9.

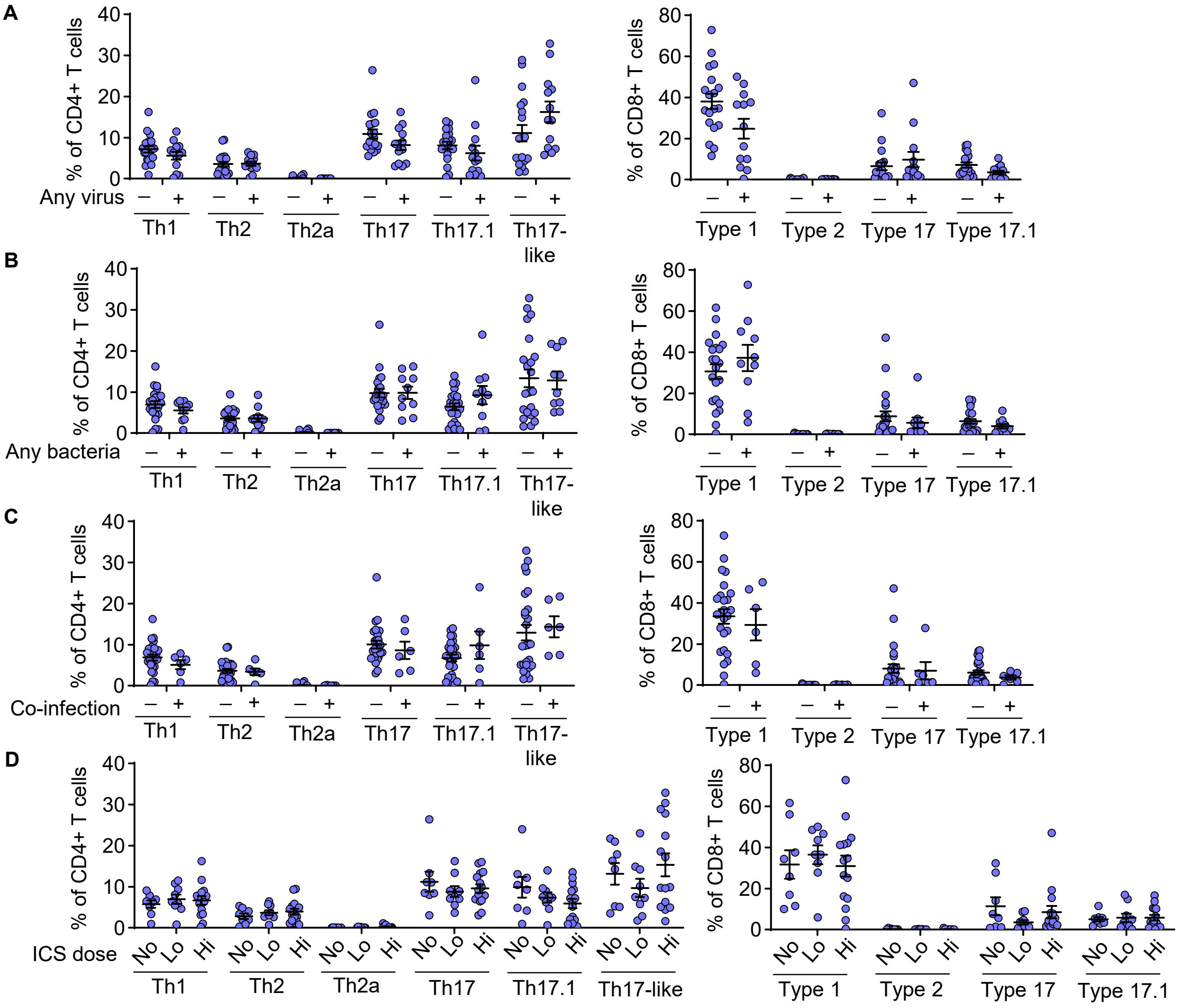

Supplemental Figure 5. Frequencies of type 1, type 2, and type 17 signatures in the airways by infection status and medication use. (A-D) Frequencies of T-cell subsets in the BAL in relation to (**A**) any virus (- n=19, + n=13), (**B**) any bacteria (- n=22, + n=10), and (**C**) co-infection (- n=26, + n=6). (**D**) Frequencies of T-cell subsets in the BAL of patients on no ICS (n=8), low dose ICS (n=9; Lo), and high dose ICS (n=15; Hi). Mean ± SEM. Multiple Mann-Whitney tests with Holm-Sidak correction (**A-C**) and Kruskal-Wallis test with Dunn's multiple comparisons test (**D**).

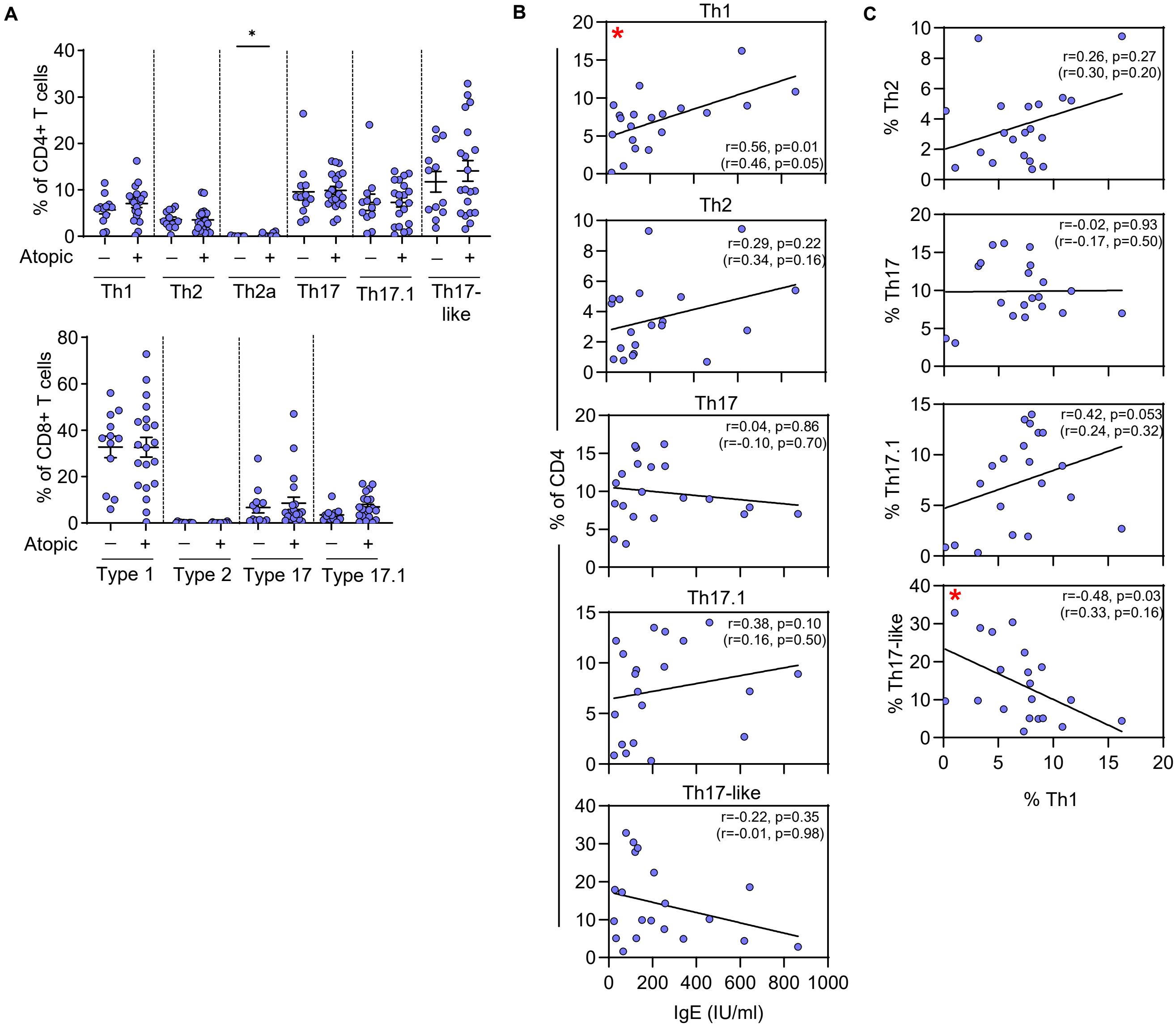

Supplemental Figure 6. Th1 cells are linked to total IgE in atopic children. (**A**) Frequencies of type 1, type 2, and type 17 subsets in the BAL of patients with (n=20) and without (n=12) atopy as defined by detection of specific IgE to at least one allergen. Mean ± SEM. (**B**) Spearman correlations between levels of total IgE and frequencies of CD4+ T-cell subsets in atopic patients. (**C**) Spearman correlations between frequencies of Th1 cells and other CD4+ subsets in atopic patients. Lines denote linear regression and red asterisks denote significant correlations. Multiple Mann-Whitney tests with Holm-Sidak correction (**A**). Values in parentheses corrected for age (**B** and **C**). *p<0.05

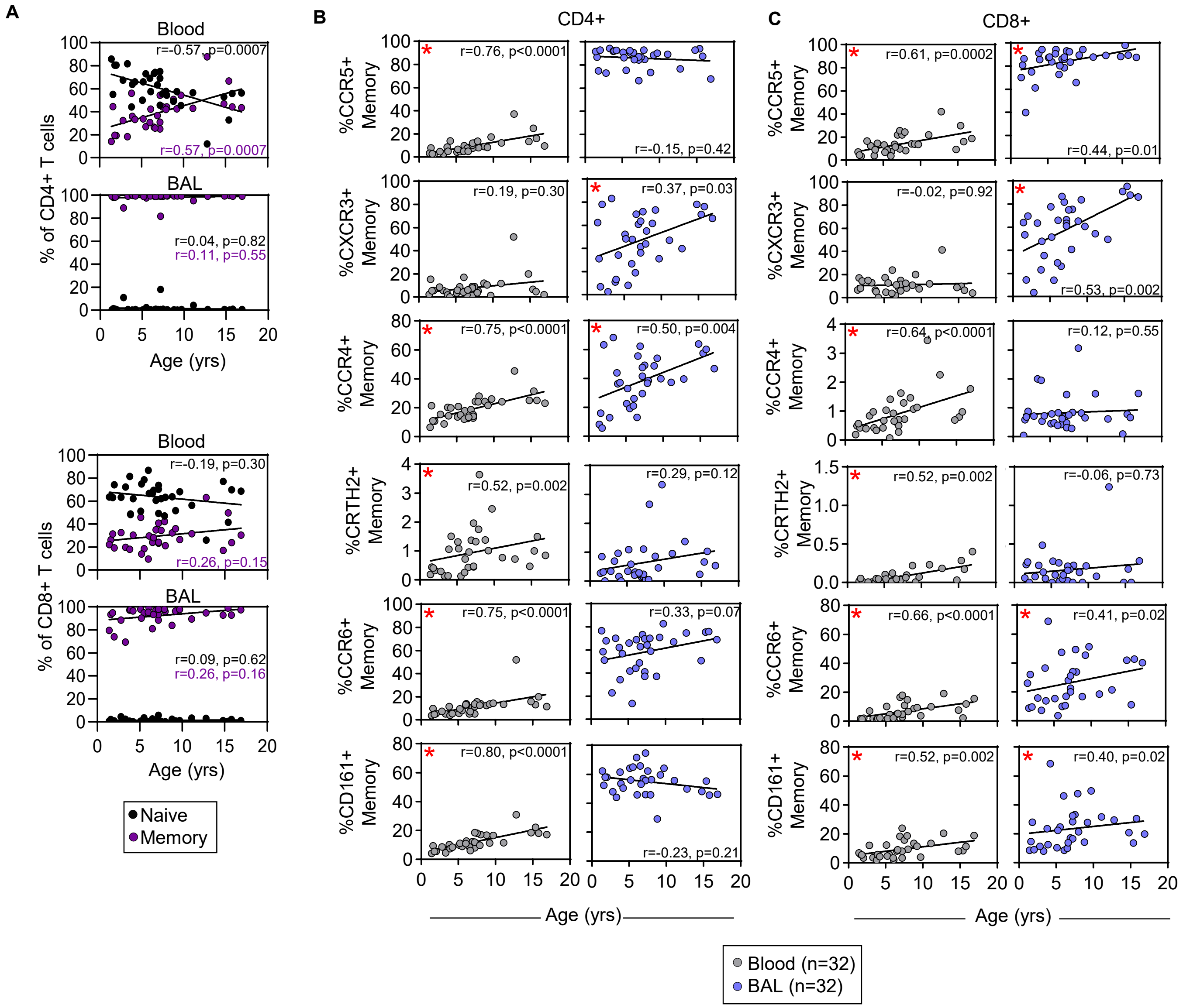

Supplemental Figure 7. Preferential accumulation of type 1 T cells in the airways with age. (**A**) Spearman correlations between age and frequencies of naïve and memory (T_{CM}, T_{EM}, TEMRA-like) CD4+ and CD8+ T cells in blood and BAL from 32 children. (**B** and **C**) Spearman correlations between age and frequencies of marker-positive memory CD4+ T cells (**B**) and CD8+ T cells (**C**) in blood and BAL. Lines denote linear regression and red asterisks denote significant correlations.

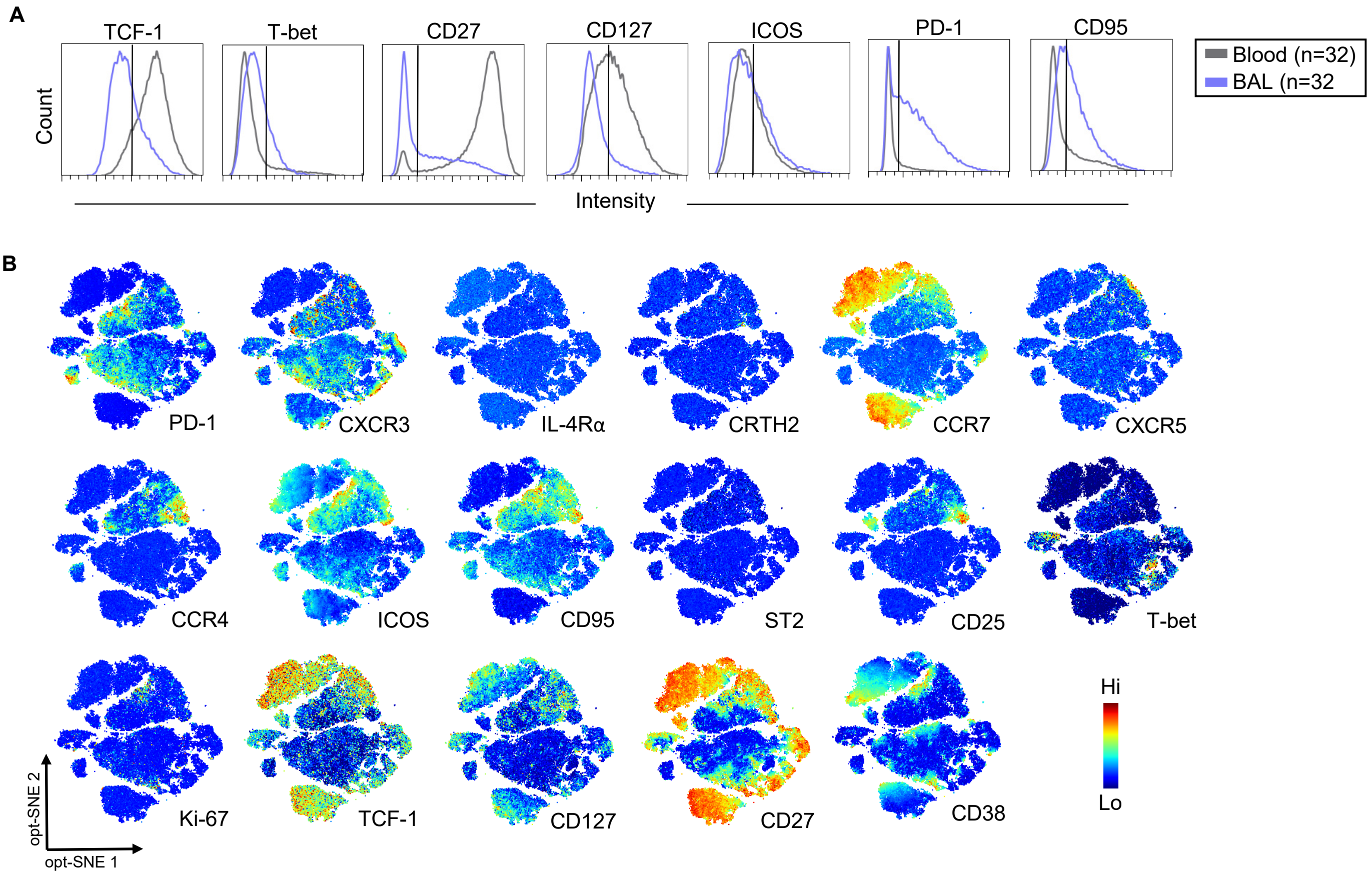

Supplemental Figure 8. Expression profile of T cells in the blood and BAL of children with recurrent wheeze. (**A**) Histograms showing the expression of select markers on blood and BAL CD3+ cells. (**B**) Expression of select phenotypic markers across all samples (n=64).

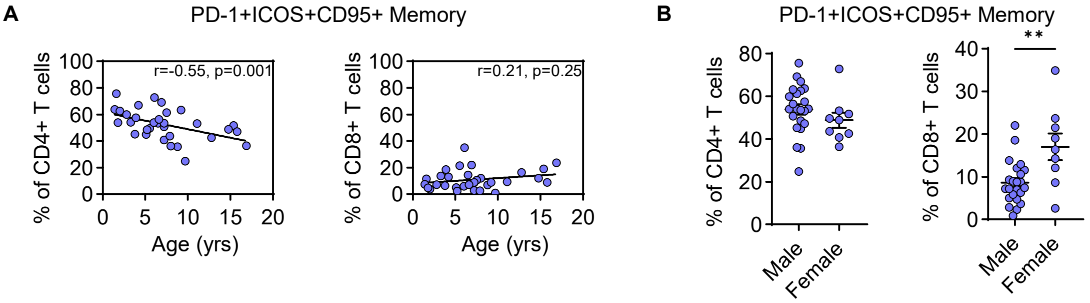

Supplemental Figure 9. Activated T cells in the lower airways by age and sex. (**A**) Spearman correlations between age and frequencies of activated CD4+ and CD8+ memory T cells in the BAL (n=32). Lines denote linear regression. (**B**) Frequencies of activated CD4+ and CD8+ memory T cells in the BAL of males (n=23) and females (n=9). Mean ± SEM. Mann-Whitney test. **p≤0.01

Supplemental Table 2. Demographic and clinical data of RV- and RV+ patients

	RV-	RV+	P value
n	22	10	N/A
Age (yrs)[A]	6.7 (5.0-8.9)	4.8 (3.0-7.7)	0.19[D]
Male sex, n (%)	16 (72.7)	7 (70.0)	>0.99[E]
White, n (%)	16 (72.7)	8 (80.0)	>0.99[E]
Age Category			0.35[E]
Pre-school (1-5yrs)	8 (36.4)	4 (40.0)	
School-age (6-11yrs)	9 (40.9)	6 (60.0)	
Teen-age (12-17yrs)	5 (22.7)	0 (0.0)	
Total IgE (IU/ml)[A]	72.7 (33.9-156.0)	43.5 (14.8-128.1)	0.35[D]
Atopic, n (%)	15 (68.2)	5 (50.0)	0.44[E]
Medication, n (%)			
Inhaled corticosteroid (ICS)	16 (72.7)	8 (80.0)	>0.99[E]
Long-acting β-agonist	13 (59.1)	1 (10.0)	0.02[E]
Antileukotriene	7 (31.9)	2 (20.0)	0.68[E]
Oral prednisone	2 (9.1)	1 (10.0)	>0.99[E]
Dupilumab	2 (9.1)	0 (0.0)	>0.99[E]
Mepolizumab	1 (4.5)	0 (0.0)	>0.99[E]
Omalizumab	1 (4.5)	0 (0.0)	>0.99[E]
BAL composition %[B]		(n=9)	
Eosinophils	0 (0-4)	0 (0-14)	0.43[D]
Neutrophils	2.5 (0-68)	29 (0-90)	0.03[D]
Macrophage	75.5 (0-95)	47 (0-88)	0.05[D]
Lymphocytes	4 (0-11)	3 (0-9)	0.60[D]
Infection status, n (%)			
Rhinovirus	0 (0.0)	10 (100.0)	<0.0001[E]
Other virus[C]	3 (13.6)	0 (0.0)	0.53[E]
Any bacteria	6 (27.3)	4 (40.0)	0.68[E]
Co-infection	2 (9.1)	4 (40.0)	0.06[E]

[A] Geometric mean (95% CI); [B] Median (range); [C] Adenovirus and Metapneumovirus; [D] Mann-Whitney test; [E] Fisher's exact test.

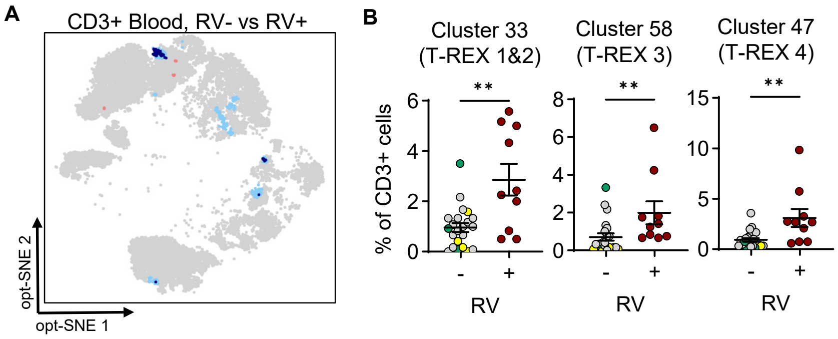

Supplemental Figure 10. T-REX analysis of blood, and frequencies of PhenoGraph clusters containing T-REX populations in RV+ and RV- children. (A) T-REX analysis of T cells comparing RV- (n=22) and RV+ (n=10) groups. (**B**) Frequencies of PhenoGraph clusters containing T-REX populations in RV- and RV+ groups. Colored symbols denote patients positive for other viruses (green, n=3), RV+ patients (red, n=10), patients negative for any virus (grey, n=15) and patients who received a biologic (yellow, n=4). Mean ± SEM. Mann-Whitney test.

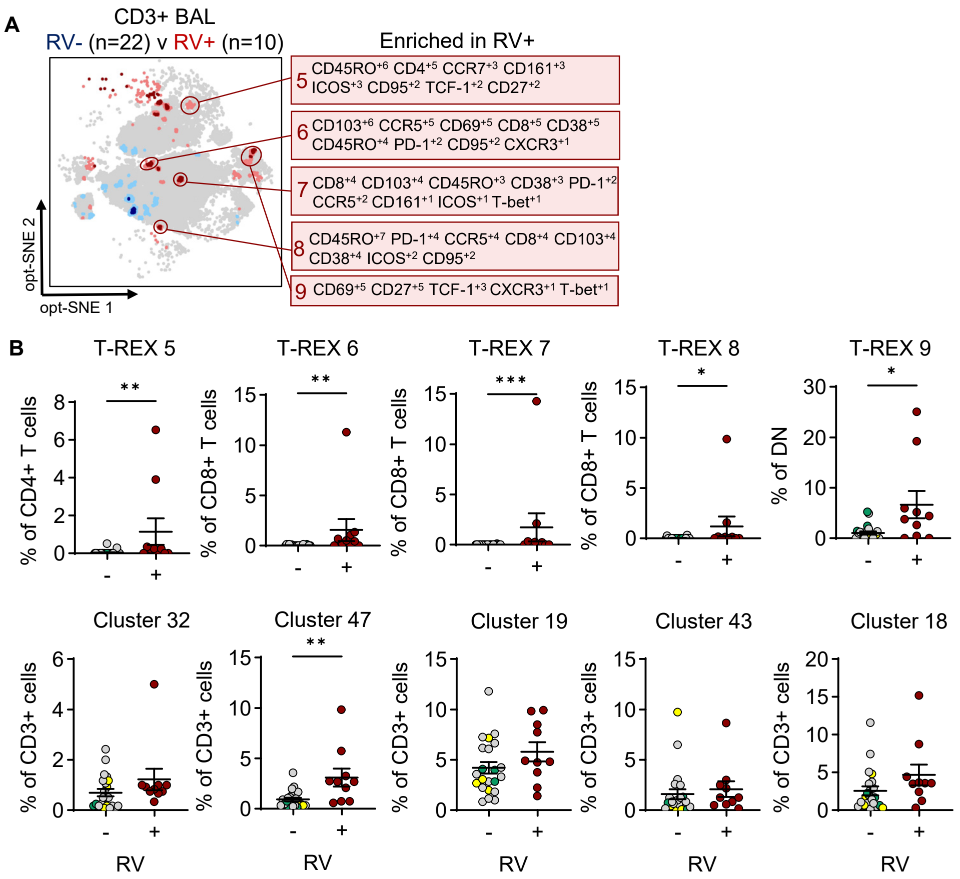

Supplemental Figure 11. "Highly variable" RV-related T-cell signatures. (**A**) MEM signatures of "highly variable" populations enriched in the RV+ group. (**B**) Frequencies of "highly variable" T-REX populations enriched in the RV+ group and their corresponding PhenoGraph clusters. Colored symbols denote patients positive for other viruses (green, n=3), RV+ patients (red, n=10), patients negative for any virus (grey, n=15) and patients who received a biologic (yellow, n=4). Mean ± SEM. Mann-Whitney test. *p<0.05, **p≤0.01, ***p≤0.001.

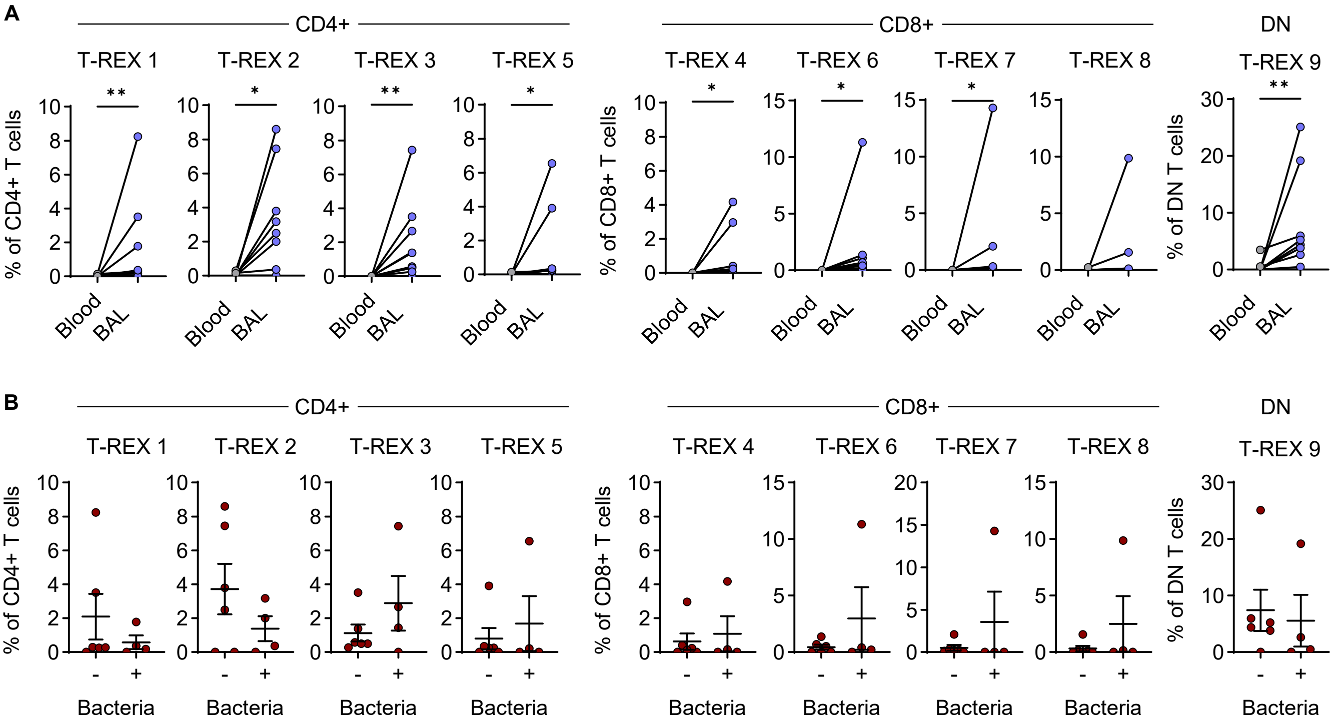

Supplemental Figure 12. Analysis of frequencies of RV-related T-REX populations 1-9. (A) Frequencies of T-REX populations in matched blood and BAL of RV+ patients (n=10). **(B)** Frequencies of T-REX populations in RV+ children who tested negative (n=6) or positive (n=4) for bacteria. Mean ± SEM. Wilcoxon matched-pairs signed rank test (**A**) and Mann-Whitney test (**B**). *p<0.05, **p≤0.01.

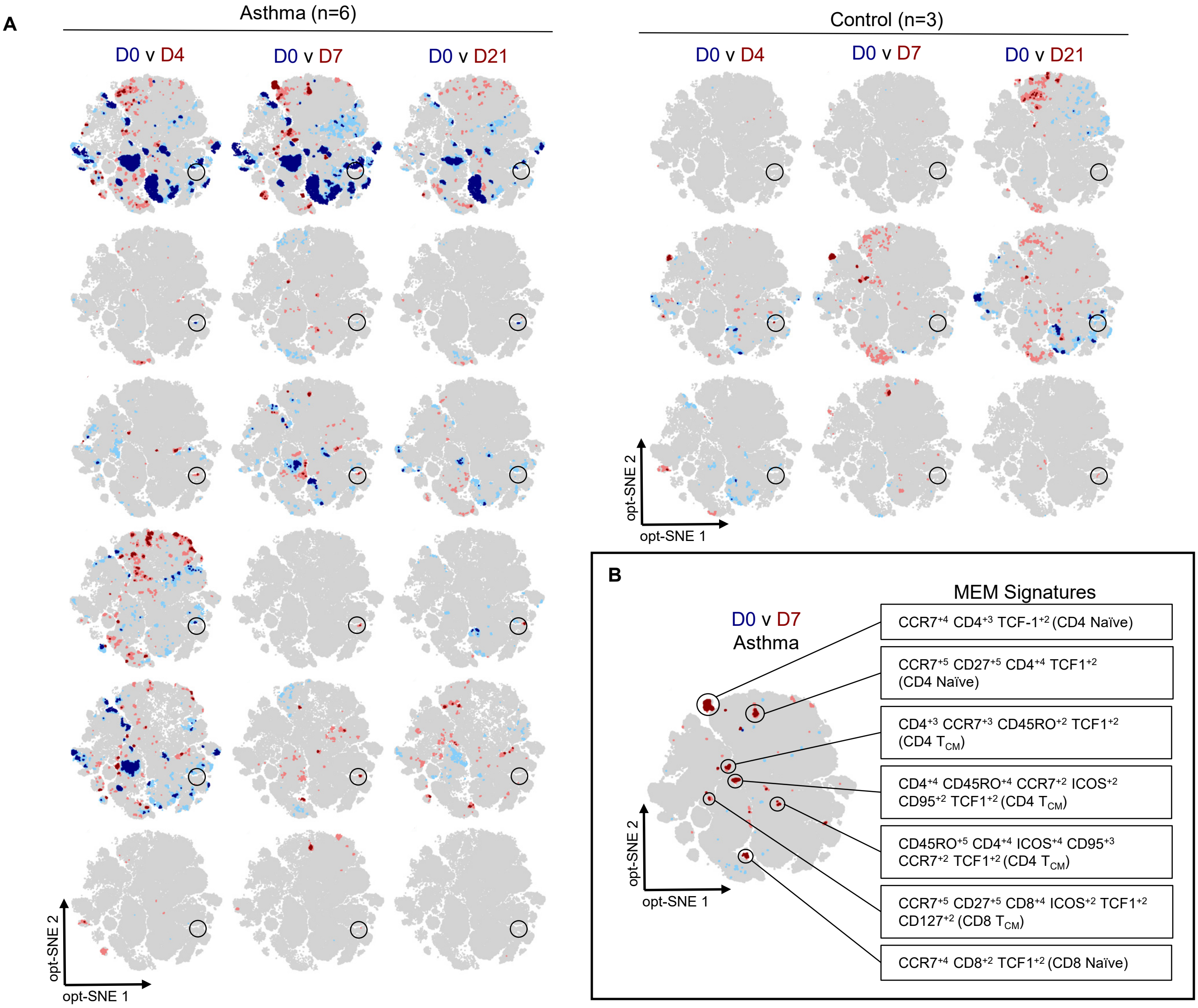

Supplemental Figure 13. Variability in the T-cell response to RV challenge. (**A**) T-REX plots for individual subjects (asthma, n=6 and healthy controls, n=4) comparing changes on days 4, 7, and 21 with day 0 of RV challenge. Black circles denote T-REX A. (**B**) "Highly variable" T-REX populations induced by RV in asthma (day 7 versus day 0).

Supplemental Table 3. Characteristics of RV-challenge study participants.

	**Asthma**	**Control**	**P value**
n	6	3	N/A
Age (yrs)[A]	21.5 (18.6-24.8)	21.3 (18.6-24.4)	0.76[B]
Male sex, n (%)	2 (33.3)	1 (33.3)	>0.99[C]
White, n (%)	4 (66.7)	3 (100.0)	0.50[C]
Total IgE (IU/ml)[A]	1139 (473.7-2737)	9.7 (1-96.4)	0.02[B]

[A] Geometric mean (95% CI); [B] Mann-Whitney test; [C] Fisher's exact test.

Supplemental Table 4. Demographics and clinical data for patients included in the intracellular cytokine analysis.

	**Cytokine profiling**	**T-cell phenotyping**	**P value**
n	6	32	N/A
Age (yrs)[A]	8.6 (6.3-11.5)	6.0 (4.7-7.6)	0.17[C]
Male sex, n (%)	5 (83.3)	23 (71.9)	>0.99[D]
White, n (%)	3 (50.0)	24 (75.0)	0.64[D]
Total IgE (IU/ml)[A]	78.2 (10.9-559.0)	61.9 (34.1-112.3)	0.82[C]
Atopic, n (%)	4 (66.7)	20 (62.5)	>0.99[D]
Medication, n (%)			
Inhaled corticosteroid (ICS)	5 (83.3)	24 (75.0)	>0.99[D]
Long-acting β-agonist	5 (83.3)	14 (43.8)	0.08[D]
Antileukotriene	2 (33.3)	9 (28.1)	>0.99[D]
Oral prednisone	0 (0.0)	3 (9.4)	>0.99[D]
Dupilumab	1 (16.7)	2 (6.3)	0.41[D]
Mepolizumab	1 (16.7)	1 (3.1)	0.29[D]
Omalizumab	0 (0.0)	1 (3.1)	>0.99[D]
BAL phenotype, n (%)		(n=31)	0.75[D]
Isolated eosinophilia	0 (0.0)	2 (6.5)	
Isolated neutrophilia	1 (16.7)	9 (29.0)	
Mixed granulocytes	1 (16.7)	2 (6.5)	
Pauci-granulocytic	4 (66.7)	18 (58.1)	
Infection status, n (%)			
Rhinovirus	0 (0.0)	10 (31.3)	0.17[D]
Other virus[B]	0 (0.0)	3 (9.4)	>0.99[D]
Any bacteria	0 (0.0)	10 (31.3)	0.17[D]
Co-infection	0 (0.0)	6 (18.8)	0.56[D]

[A] Geometric mean (95% CI). [B] Adenovirus and Metapneumovirus. [C] Mann-Whitney test. [D] Fisher's exact test.

Supplemental Table 5. Spectral flow cytometry panel for intracellular cytokine analysis.

Marker	Color	Clone	Vendor	Catalog number	Dilution
Viability	Live/Dead Blue		Invitrogen	L23105	1:4000
CD3	BUV395	SK7	BD	564001	1:50
CD4	BUV496	OKT4	BD	750980	1:50
CD45RO	BUV563	UCHL1	BD	748369	1:100
CXCR3	BUV661	1C6/CXCR3	BD	741649	1:50
CCR5	BUV737	2D7/CCR5	BD	612808	1:25
CD69	BUV805	FN50	BD	748763	1:100
CD14	BV510	M5E2	Biolegend	301842	1:20
CD19	BV510	HIB19	Biolegend	302242	1:20
IL-4	BV605	MP4-25D2	Biolegend	500828	1:40
CD103	BV711	Ber-ACT8	Biolegend	350221	1:50
TNF-α	BV750	MAb11	BD	566359	1:200
IL-17A	BV785	BL168	Biolegend	512338	1:100
IL-13	FITC	B-P6	Invitrogen	BMS133FI	1:50
CD8	PerCP	SK1	Biolegend	344708	1:200
Granzyme B	RB705	GB11	BD	570275	1:100
IL-21	PE	3A3-N2.1	BD	562042	1:50
CD161	PE-Dazzle 594	HP-3G10	Biolegend	339940	1:20
IL-2	PE-Cy7	MQ1-17H12	BD	560707	1:50
IL-5	APC	JES1-39D10	Miltenyi Biotec	130-127-364	1:20
IFN-γ	Alexa Fluor 700	4S.B3	Biolegend	502520	1:1000
IL-22	APC-Fire 750	2G12A41	Biolegend	366713	1:50

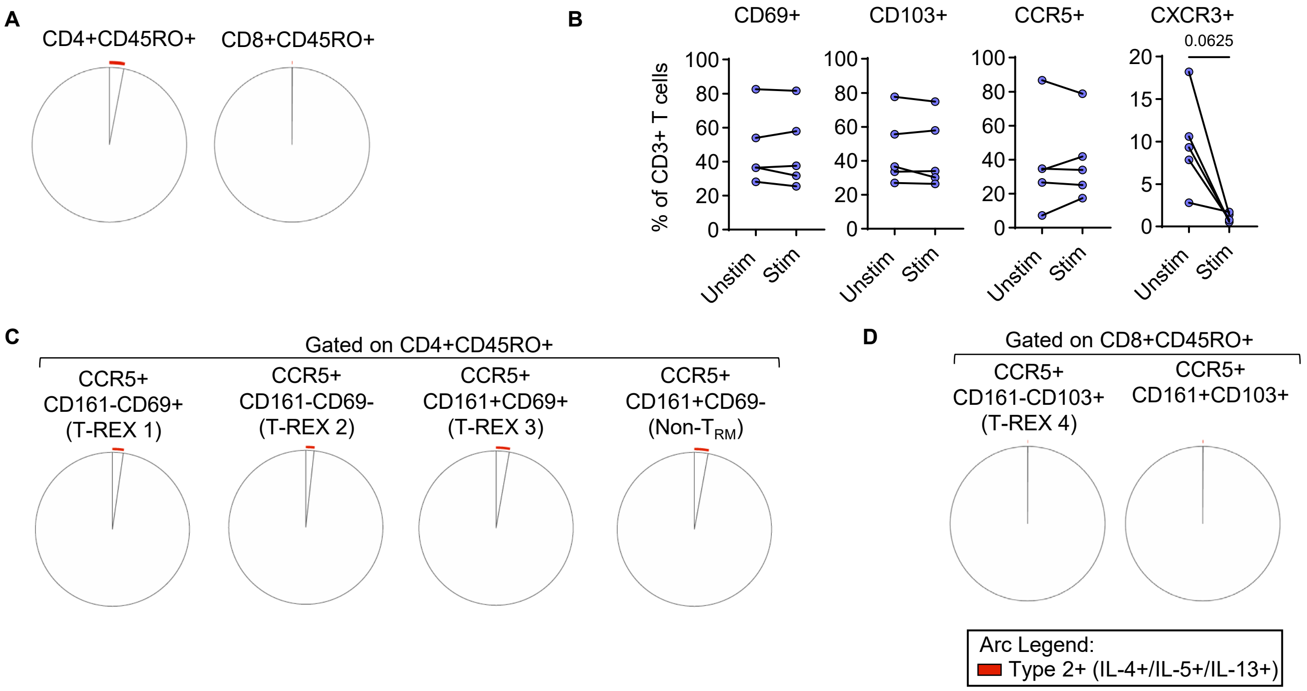

Supplemental Figure 14. Type 2 cytokine production is minimal in the lower airways of children with recurrent wheeze. (**A**) SPICE plots showing average type 2 cytokine signatures in CD4+ and CD8+ memory T cells in the BAL (n=6). (**B**) Change in marker expression after stimulation of BAL cells (n=5). Mean ± SEM. (**C** and **D**) Average type 2 cytokine signatures in candidate CD4+ and CD8+ T-REX populations (n=6). Wilcoxon matched-pairs signed rank test (**B**).

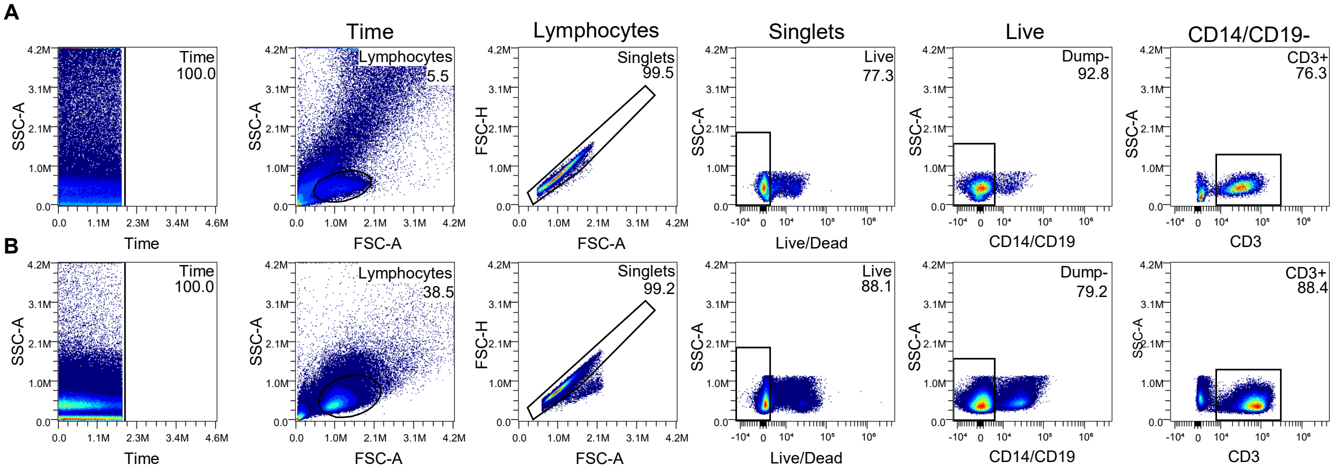

Supplemental Figure 15. Representative scatter plots showing manual gating strategy for pre-processing of (**A**) blood and (**B**) BAL samples.

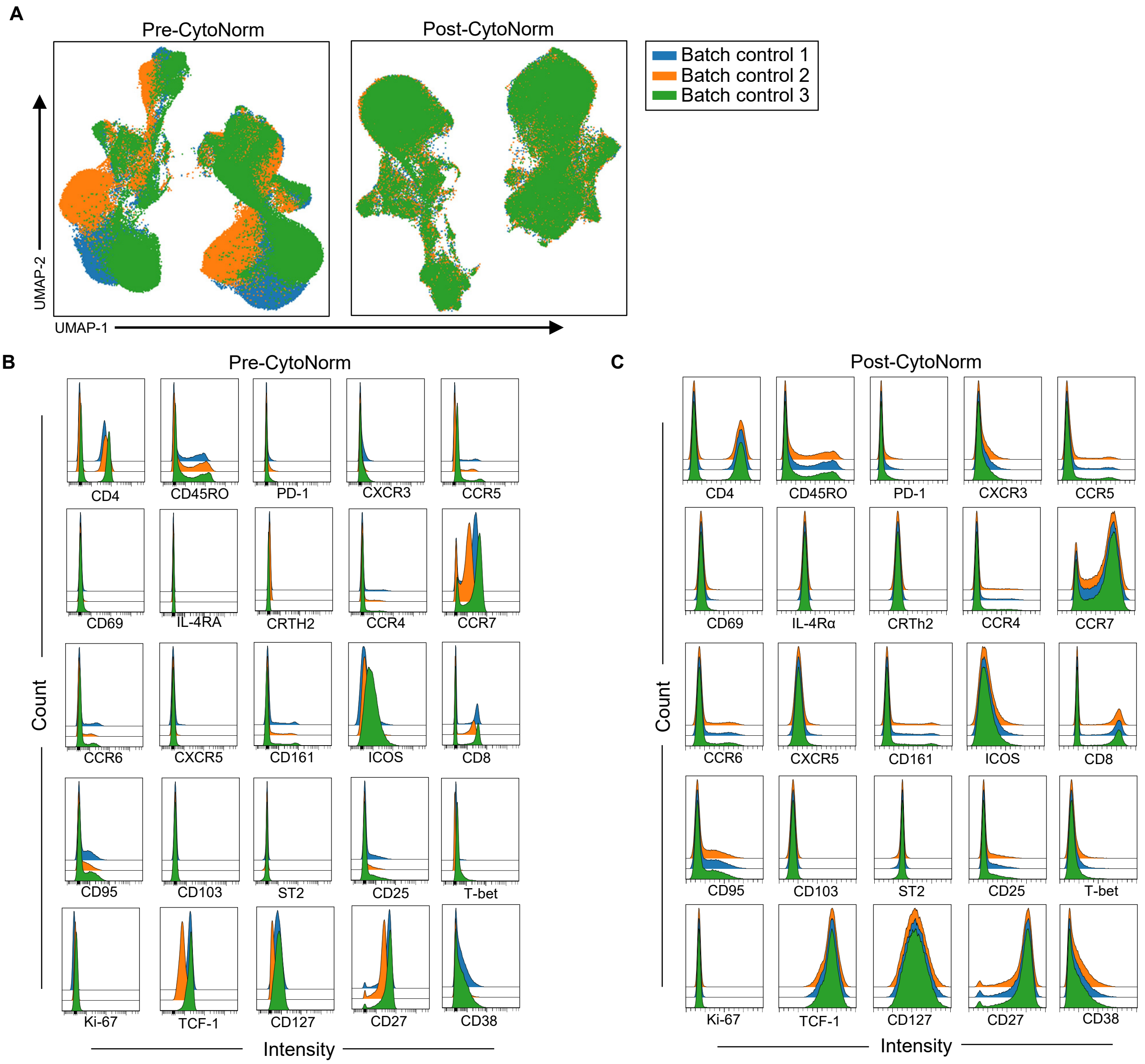

Supplemental Figure 16. Results of CytoNorm data normalization. (**A**) UMAP of batch control samples, pre- and post-normalization using Cytonorm. Histograms showing the distribution of each marker within each batch control, (**B**) pre- and (**C**) post-normalization.
